# Correcting Structural Bias in Dynamical Models of Infectious Disease Using a Bayesian State-Space Framework

**DOI:** 10.1007/s11538-026-01730-3

**Published:** 2026-08-11

**Authors:** Miracle Amadi, José Carlos García-Merino, Heikki Haario

**Affiliations:** 1 LUT School of Engineering Sciences, Lappeenranta-Lahti University of Technology (LUT), Yliopistonkatu 34, Lappeenranta, Finland; 2 Department of Mathematics, School of Technology, 10003 Cáceres, Spain

**Keywords:** Malaria transmission, Dynamical systems, Stochastic differential equations, Bayesian state-space models, Hamiltonian Monte Carlo, Model discrepancy

## Abstract

Deterministic dynamical models are widely used in infectious disease modelling, but they often become systematically biased when key drivers such as seasonality and random environmental variation are simplified or omitted. This paper proposes a practical way to account for structural bias while preserving the underlying mechanistic model. We model the observed time series as the sum of (i) a deterministic transmission component given by a reduced Ross malaria model and (ii) a latent stochastic seasonal component that captures unresolved seasonal forcing and other unmodelled variability. The seasonal component is defined as a mean-reverting stochastic differential equation with periodic forcing, which can be interpreted as a seasonally forced Ornstein–Uhlenbeck (OU) process and, at the same time, as a dynamically constrained model-discrepancy term. The combined model forms an additive Bayesian state-space system. A key challenge in additive decompositions is identifiability: many combinations of the deterministic and stochastic components can explain the same observations. We therefore use informative priors to stabilise this decomposition, together with a non-centred parameterisation (a reparameterisation that improves MCMC efficiency by sampling standardised noise terms instead of states directly) of the latent stochastic differential equation (SDE) states that enables efficient joint inference with Hamiltonian Monte Carlo (NUTS) in Stan. We validate the approach in three steps: (1) an OU example that contrasts parameter-only inference with latent state-space inference, (2) synthetic experiments that have a good agreement with the observed signal while highlighting the expected negative posterior dependence between components, and (3) an application to five years of monthly malaria case reports from Delta State, Nigeria. In the real-data analysis, the proposed additive ODE–SDE model produces a close fit with coherent uncertainty quantification and a flexible seasonal reconstruction, while keeping the mechanistic transmission model interpretable. Overall, the framework provides a flexible and transferable method for accounting for structural model discrepancy in misspecified dynamical models using a structured stochastic discrepancy.

## Introduction

Mathematical models are essential tools for understanding the dynamics of infectious diseases and guiding public health interventions (Anderson and May 1991; Brauer et al. 2008; Keeling and Rohani 2008). Many classical epidemiological models are formulated as deterministic systems of differential equations, providing a tractable framework for analysing transmission dynamics and evaluating control strategies (Anderson and May 1991; Brauer et al. 2008; Keeling and Rohani 2008; Ruan 2006). However, these models often rely on simplifying assumptions, such as deterministic and time-invariant structures, that limit their ability to capture the complexity of real-world epidemiological patterns. Such assumptions can lead to *structural bias*, where a model systematically misrepresents key aspects of system behaviour even after parameter calibration (Spanos 2011). In particular, the assumption of smooth deterministic evolution often fails to account for the stochasticity and seasonal variability observed in many infectious disease systems, including malaria transmission dynamics (Grassly and Fraser 2006; Allen 2017).

Malaria transmission is strongly influenced by environmental drivers such as rainfall and temperature, which affect mosquito population dynamics and vector–host interactions (Smith et al. 2012; Macdonald 1957). These factors introduce pronounced seasonal patterns and random fluctuations that cannot be adequately represented by static models or simple seasonal forcing terms. When such dynamics are omitted or oversimplified, model predictions may become systematically biased and unreliable.

To illustrate this issue, we consider the classical Ross model, one of the foundational models in malaria transmission research (Ross 1911; Macdonald 1957; Smith et al. 2012). The Ross model provides a useful starting point because of its conceptual simplicity and historical importance in epidemiology. However, in its basic form it assumes constant mosquito density and therefore lacks the flexibility to capture seasonal or nonlinear variations in mosquito dynamics. As a result, it offers a clear example of how structural simplifications in mechanistic models can lead to systematic discrepancies between model predictions and observed epidemiological dynamics.

Two broad approaches are commonly used to account for stochasticity in mechanistic epidemic models. One approach is to replace the deterministic formulation with a fully stochastic epidemic model. Another approach retains the deterministic skeleton while introducing stochasticity into selected model components, for example by allowing transmission rates to evolve as latent diffusion processes within an otherwise deterministic framework (Dureau et al. 2013). In line with the latter alternative of introducing temporal variation into one or a small number of model quantities, seasonal effects are frequently represented through sinusoidal forcing functions that modify transmission parameters, often motivated by the relationship between mosquito density and rainfall (Chitnis et al. 2012; Childs and Boots 2009; Prosper et al. 2023).

In our earlier work (Amadi and Haario 2023), we adopted a related strategy using a Kalman filter framework, in which mosquito density was treated as a time-varying latent state and estimated jointly with other system variables through state augmentation techniques (Kitagawa 1998). Although effective in some cases, this approach required careful calibration of the model and observation noise covariances, and its performance was sensitive to structural assumptions and data quality (Hakkarainen et al. 2012; Solonen and Järvinen 2013; Solonen et al. 2014). Like other approaches in this class, it requires identifying where stochasticity should be incorporated within the mechanistic model. However, malaria seasonality arises from the combined influence of climate variability, vector ecology, human behaviour, and immunity dynamics (Grassly and Fraser 2006). Thus, attributing unresolved variability to a single mechanistic component may oversimplify the underlying mechanisms and limit model realism.

In the present work, we retain the deterministic transmission model but augment it with an additive latent stochastic process to represent unresolved variability. Specifically, we model the observed malaria cases as the sum of a deterministic transmission component derived from the Ross model and a stochastic process governed by a stochastic differential equation (SDE). Unlike approaches that introduce stochasticity into selected mechanistic parameters or states, the additive process accounts for seasonal variability and structural model error while leaving the underlying transmission model unchanged, thereby preserving its interpretability. As a result, the framework can be incorporated into an existing deterministic model without reformulating its governing equations.

Conceptually, this formulation is closely related to the discrepancy framework of Kennedy and O’Hagan, in which structural model error is represented through an explicit discrepancy term that accounts for systematic differences between a mechanistic model and the real system (Kennedy and O’Hagan 2001; Brynjarsdóttir and O’Hagan 2014). In our formulation, this role is played by the latent seasonal process, which captures unobserved seasonal variability and random fluctuations without imposing restrictive assumptions about their origin. Although illustrated here for malaria transmission, the framework is general and can be applied to other dynamical systems whose mathematical models are affected by structural bias.

Parameter and state estimation are performed jointly using *Bayesian inference*, implemented via Hamiltonian Monte Carlo (HMC) with a non-centred parameterization. Hamiltonian Monte Carlo provides an efficient approach for exploring high-dimensional posterior distributions and forms the basis of modern probabilistic programming frameworks such as Stan (Hoffman et al. 2014; Carpenter et al. 2017). The non-centred parameterization further improves sampling efficiency by reducing posterior dependencies between latent states and scale parameters (Papaspiliopoulos et al. 2007; Betancourt and Girolami 2015). Together, these techniques provide coherent uncertainty quantification for both deterministic and stochastic components of the model.

The main contributions of this work are threefold. First, we introduce an additive ODE–SDE framework that explicitly separates deterministic transmission dynamics from unresolved stochastic seasonal variability. Second, we formulate the resulting model as a Bayesian state–space system with joint inference of parameters and latent states. Third, we demonstrate how non-centered parameterizations enable efficient inference for such models using Hamiltonian Monte Carlo. The proposed methodology is first validated on synthetic data and then applied to malaria case reports from Nigeria, demonstrating how it accounts for structural bias and improves predictive realism. Some aspects of the synthetic identifiability experiments were previously reported in an earlier preliminary study (Amadi and Haario 2026). The present paper extends that work by providing a fuller methodological treatment, analysing the identifiability of the latent decomposition between deterministic and stochastic components, and applying the framework to real malaria case data.

The remainder of the paper is organised as follows. Section 2 introduces the proposed additive ODE-SDE model, including the stochastic seasonal component and its Bayesian state-space formulation. Section 3 presents validation experiments on synthetic data and the application to malaria case data, together with posterior diagnostics and uncertainty quantification. Section 4 summarises the main findings and discusses implications, limitations, and directions for future work.

## Methods

This section presents the deterministic and stochastic components of the model, outlines their mathematical formulations, and describes the statistical structure used for inference.

### Deterministic Component: The Ross Model

As the deterministic backbone, we consider the classical Ross model, which describes the time evolution of the fractions of infected humans and mosquitoes, denoted $i_h$ and $i_m$, respectively. The model is defined by two coupled differential equations:

1 $$\begin{aligned} \frac{di_h}{dt}&= m a b\, i_m (1 - i_h) - r i_h, \\ \frac{di_m}{dt}&= a c\, i_h (1 - i_m) - \mu i_m, \nonumber \end{aligned}$$

where *m* is the mosquito-to-human ratio, *a* the mosquito biting rate, *b* and *c* the probabilities of transmission from mosquito to human and vice versa, μ the mosquito mortality rate, and *r* the human recovery rate (Ross 1910).

**Fig. 1:**
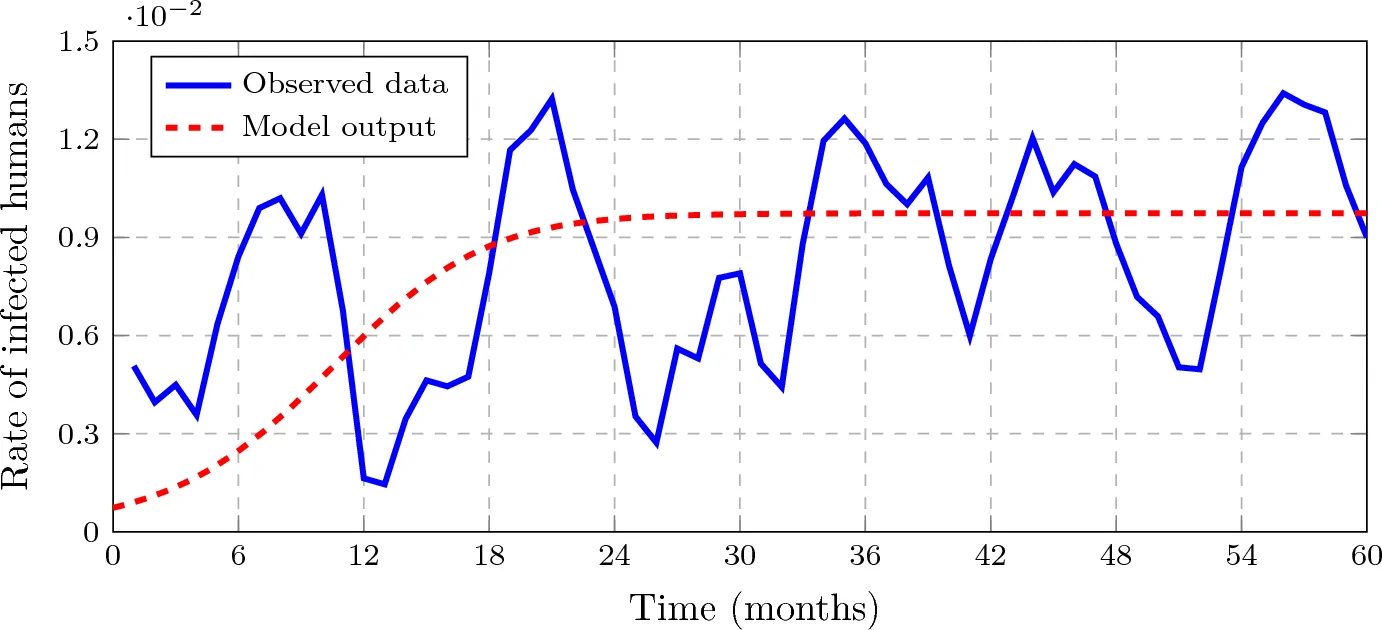
Comparison between observed malaria cases and simulated trajectories from the classical Ross model (color figure online)

The Ross model has played a central role in malaria transmission research and has strongly influenced the development of control strategies. However, it omits important features of real-world dynamics. For instance, the mosquito density parameter *m* is treated as constant, whereas in reality it varies with environmental conditions such as rainfall and temperature (Asgarian et al. 2021). Similarly, other parameters may exhibit seasonal variation or nonlinear dependencies that are difficult to quantify. Figure 1 compares a simulated Ross model trajectory with observed malaria case data, illustrating the structural gap between the deterministic model and empirical observations.

To tailor model complexity to the information content of the data, one practical approach is to reduce model dimensionality so that parameters correspond more directly to measurable system quantities (Wieland et al. 2021). Since mosquito dynamics evolve on a faster timescale than human infection, the infected mosquito proportion can be approximated by its quasi-equilibrium value (Rocha et al. 2013; Segel and Slemrod 1989):

2 $$\begin{aligned} i_m^* = \frac{i_h}{i_h + \kappa }, \qquad \text {where } \kappa = \frac{\mu }{a c}. \end{aligned}$$

Substituting this expression into the human infection equation yields a reduced form of the Ross model:

3 $$\begin{aligned} \frac{di_h}{dt} = m a b\, \frac{i_h}{i_h + \kappa } (1 - i_h) - r i_h. \end{aligned}$$

This reduced model retains the key biological mechanisms while reducing the number of parameters to be estimated. Following (Amadi and Haario 2022), three parameters are treated as identifiable (see also Appendix A .2): the mosquito-to-human transmission intensity β=mab, the human recovery rate *r*, and the mosquito infection threshold $\kappa = \mu / (a c)$. Thus, the parameter vector is $\theta = \{\beta , \kappa , r\}$.

While this deterministic model captures the mean behaviour of the system, it cannot reproduce the irregular or periodic fluctuations typically observed in real data. To address this, we augment the model with a stochastic component that accounts for unmodelled external variability and seasonal effects.

### Stochastic Seasonal Component

To represent fluctuations that the deterministic ODE cannot explain, we introduce a stochastic seasonal process. The idea is intuitive: malaria incidence, like many ecological and epidemiological systems, exhibits recurring seasonal cycles superimposed with irregular random variation (Roca-Feltrer et al. 2009). We capture this behaviour through a latent process $S_t$ that combines a periodic deterministic driver with random noise. This latent process can also be interpreted within the model discrepancy framework of Kennedy and O’Hagan (2001), where a bias term $\delta (\cdot )$ is introduced to represent systematic deviations between model output and observations. In our formulation, $S_t$ given in Eq. 4 plays the role of this discrepancy function, capturing the components of variability not explained by the deterministic Ross model. However, unlike the unconstrained Gaussian process typically used in that framework, our discrepancy term is dynamically constrained by a stochastic differential equation, ensuring temporal coherence and interpretable seasonal structure.

The process $S_t$ evolves according to a mean-reverting stochastic differential equation (SDE),

4 $$\begin{aligned} dS_t = \left( \alpha \cos \!\left( \frac{2\pi t}{T} \right) - \frac{S_t}{\ell } \right) dt + \sigma \, dW_t, \end{aligned}$$

where α controls the strength of seasonal forcing, *T* is the period of the seasonal cycle, ℓ determines the mean-reversion timescale, σ is the magnitude of stochastic fluctuations, and $W_t$ is a standard Brownian process.

This formulation can be viewed as a seasonally forced Ornstein-Uhlenbeck (OU) process (Maller et al. 2009). When α=0, it reduces to the classical OU process, which reverts to a constant mean of zero. For α>0, the mean itself oscillates periodically, producing a smooth seasonal cycle with random perturbations. This structure allows $S_t$ to represent both regular seasonal forcing and unmodelled stochastic variability. For a more detailed explanation of the relationship between the seasonal process and the classical OU formulation, see Appendix A .1.

For numerical implementation, we use the Euler–Maruyama discretisation of (4) (Kloeden and Pearson 1977; Oksendal 2013),

5 $$\begin{aligned} S_t = S_{t-1} + \left( \alpha \cos \!\left( \frac{2\pi (t-1)}{T} \right) - \frac{S_{t-1}}{\ell } \right) \! \Delta t + \sigma \sqrt{\Delta t}\, z_{t-1}, \quad z_{t-1} \sim \mathcal {N}(0,1). \end{aligned}$$

To illustrate the stochastic behaviour of the seasonal component, we simulate several independent realisations of (5) using a representative parameter setting with seasonal period T=12 and parameters $(\alpha ,\ell ,\sigma )=(0.1,\,6,\,0.05)$.

**Fig. 2:**
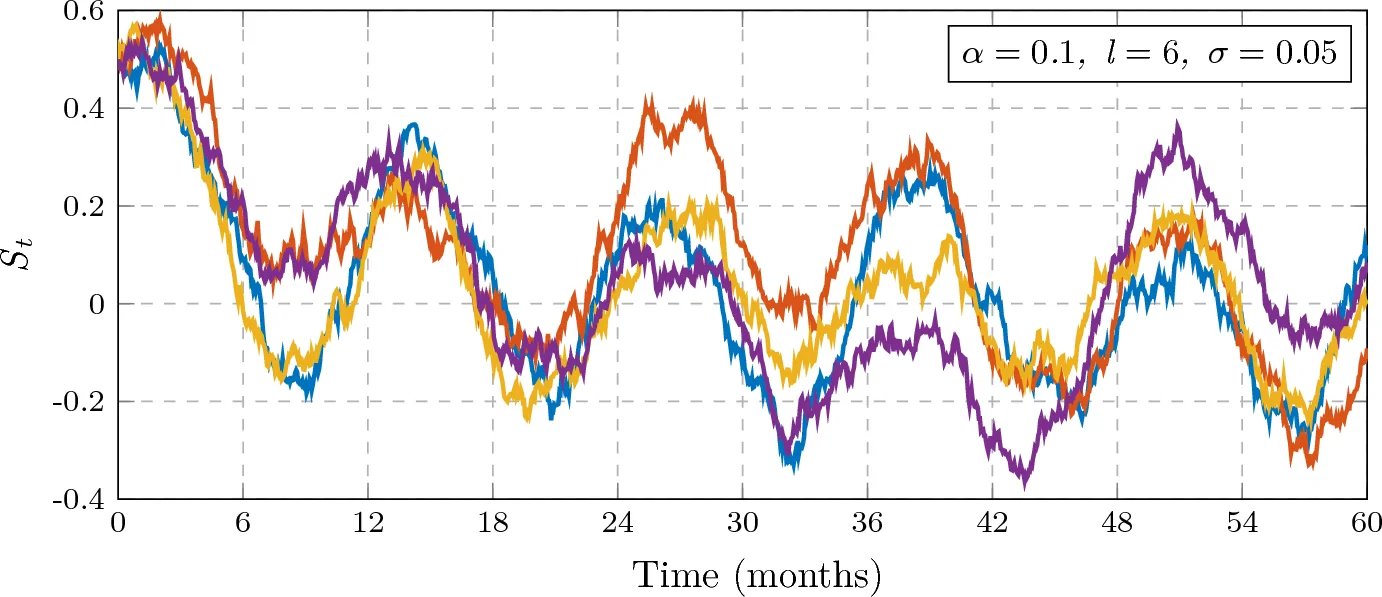
Independent realisations of the stochastic seasonal process $S_t$ generated from (5) using identical parameter values $(\alpha =0.1,\ \ell =6,\ \sigma =0.05)$. Although the parameters are fixed, the trajectories differ because of the stochastic Wiener noise term, illustrating the inherent randomness of the process (color figure online)

As shown in Fig. 2, each trajectory follows the same underlying seasonal pattern but exhibits distinct fluctuations due to stochastic forcing. This illustrates a fundamental property of stochastic differential equation models: the parameters determine the statistical structure of the process, while individual trajectories remain random. Consequently, the latent process cannot be uniquely determined from the parameters alone. This inherent uncertainty motivates the joint estimation of both model parameters and latent states within a probabilistic framework. Also, see additional simulations illustrating how the parameters α, ℓ, and σ influence the dynamics of the seasonal process are provided in Appendix C .2.

### Joint Additive Formulation for ODE–SDE Components

Having specified the deterministic ODE and the stochastic seasonal SDE components, we now combine them into a unified additive state–space formulation. Let $\theta =\{\beta ,\kappa ,r\}$ denote the ODE parameters and $\psi =\{\alpha ,\ell ,\sigma \}$ the SDE parameters. With observations at times t=1,⋯,N (sampling interval Δt), the data model is

6 $$\begin{aligned} y_t = X_t + S_t + e_t, \qquad e_t \sim \mathcal {N}(0,\sigma _e^2). \end{aligned}$$

Given $\textbf{y}=\{y_t\}_{t=1}^N$, the likelihood is

$$ p(\textbf{y}\mid \textbf{X}_{1:N},\textbf{S}_{1:N},\sigma _e^2) = \prod _{t=1}^N \mathcal {N}\!\left( y_t \mid X_t + S_t,\sigma _e^2\right) . $$

The ODE trajectory $\textbf{X}_{1:N}=(X_1,\ldots ,X_N)$ is deterministic given $X_0$ and θ, reconstructed via the Euler discretisation

$$ X_t = X_{t-1} + \Delta t\, f(X_{t-1},\theta ), \qquad t=1,\dots ,N, $$

so that $\textbf{X}_{1:N}$ is a deterministic function of $(X_0,\theta )$.

In contrast, the SDE trajectory $\textbf{S}_{1:N}$ evolves stochastically through Gaussian innovations. To improve computational efficiency, we adopt a non–centered parameterization (Papaspiliopoulos 2003; Papaspiliopoulos et al. 2007; Betancourt and Girolami 2015). In a centered parameterization, the latent states $S_t$ are sampled directly, which can induce strong posterior dependence between the innovation scale σ and the state trajectory, particularly when the noise level is small.

In the non–centered formulation, we instead introduce standardized innovations $z_{t-1}\sim \mathcal {N}(0,1)$ and reconstruct the states deterministically via the Euler–Maruyama recursion (5). Each posterior draw therefore corresponds to a different innovation vector $\textbf{z}$, which generates a different latent trajectory $\textbf{S}_{1:N}$. This reparameterization reduces posterior coupling and typically improves sampling efficiency in Hamiltonian Monte Carlo.

The state evolution for $\textbf{S}_{1:N}$ induces a Gaussian Markov prior $p(\textbf{S}_{1:N}\mid S_0,\psi )$; the state-space representation of the stochastic seasonal component is given in Appendix B.

The joint posterior over states and parameters is therefore

7 $$\begin{aligned}&p(\textbf{S}_{1:N},X_0,\theta ,\psi ,\sigma _e^2 \mid \textbf{y}) \propto p(\textbf{y}\mid \textbf{X}_{1:N},\textbf{S}_{1:N},\sigma _e^2) \, p(\textbf{S}_{1:N}\mid S_0,\psi ) \, \nonumber \\&p(X_0) \, p(\theta ) \, p(\psi ) \, p(\sigma _e^2), \end{aligned}$$

where $\textbf{X}_{1:N}$ is the deterministic image of $(X_0,\theta )$ under the ODE dynamics.

We implemented the model in Stan using this discrete-time recursive formulation. Parameters included the ODE coefficients $(\beta ,\kappa ,r)$, SDE coefficients $(\alpha ,\ell ,\sigma )$, initial states $(X_0,S_0)$, the innovation vector $\textbf{z}$, and the observation noise scale $\sigma _e$. The transformed parameters block reconstructs $\textbf{X}$ and $\textbf{S}$ at each iteration. Posterior inference uses the No-U-Turn Sampler (NUTS), Stan’s adaptive HMC algorithm (Carpenter et al. 2017; Hoffman et al. 2014).

This implementation is computationally convenient because the observations are available on a regular time grid and both components can be propagated with simple discrete-time recursions: a forward Euler update for the ODE component and an Euler–Maruyama update for the stochastic component. Priors were chosen to ensure numerical stability and help resolve weak identifiability in the additive decomposition while remaining weakly informative. Their specific forms and sensitivity analyses are reported in Sect. 3.

### Ornstein–Uhlenbeck Example: Marginal Likelihood and Latent State–Space Formulations

Before applying the framework to malaria data, we first illustrate the inference approach using an analytically tractable stochastic process: the OU model. The OU process provides a convenient benchmark because its transition density is known in closed form, allowing us to compare two alternative Bayesian formulations of the same model.

A key feature of stochastic differential equations is that the model parameters determine the statistical properties of the process but do not uniquely determine a single trajectory, since each realization also depends on unobserved stochastic noise increments. This example therefore illustrates the distinction between parameter-only inference and joint inference of both parameters and latent states, and clarifies how non-centered parameterizations facilitate efficient sampling in state-space models (Durbin and Koopman 2012; Shumway and Stoffer 2006).

We consider the zero-mean OU process

$$ dX_t = -\theta \,X_t\,dt + \sigma \,dW_t, \qquad \theta>0,\ \sigma >0, $$

where $W_t$ denotes a standard Wiener process. The OU process is a classical mean-reverting diffusion whose properties are well known (Oksendal 2013; Pavliotis 2014).

We assume the process is observed at discrete times $t_n = n\Delta t$, n=1,⋯,N. For notational simplicity we write $X_n = X(t_n)$. The OU process admits the exact discrete-time transition

8 $$\begin{aligned} X_n \mid X_{n-1},\theta ,\sigma \sim \mathcal {N}\!\Big (a\,X_{n-1},\, q\Big ), \qquad a := e^{-\theta \Delta t},\quad q := \frac{\sigma ^2}{2\theta }\bigl (1-a^2\bigr ), \end{aligned}$$

with stationary variance $\textrm{Var}(X_t)=\sigma ^2/(2\theta )$ (Pavliotis 2014).

We assume observations satisfy

$$ y_n = X_n + e_n, \qquad e_n \sim \mathcal {N}(0,\sigma _e^2), $$

where $\sigma _e$ denotes the observation noise scale.

We compare two Bayesian formulations that use the same transition density (8) but differ in how the model is factorized.

**Case A: Exact Marginal Likelihood.**

In the first formulation we assume noiseless observations, $y_n \equiv X_n$, corresponding to the limit $\sigma _e = 0$. The latent states are not introduced explicitly; instead the likelihood is written directly from the OU transition density:

9 $$\begin{aligned} p(y_{1:N}\mid \theta ,\sigma ) = \underbrace{\mathcal {N}\!\Big (y_1\,\Big |\,0,\ \tfrac{\sigma ^2}{2\theta }\Big )}_{\text {stationary initial state}} \prod _{n=2}^{N} \underbrace{\mathcal {N}\!\big (y_n\,\big |\,a\,y_{n-1},\,q\big )}_{\text {OU transition}} . \end{aligned}$$

Inference in this formulation targets only the parameters $(\theta ,\sigma )$.

Given posterior draws $(\theta ^{(s)},\sigma ^{(s)})\sim p(\theta ,\sigma \mid y)$, an unconditional posterior predictive trajectory $\tilde{y}^{(s)}$ is generated by

$$ \tilde{y}^{(s)}_1 \sim \mathcal {N}\!\Big (0,\tfrac{(\sigma ^{(s)})^2}{2\theta ^{(s)}}\Big ), \qquad \tilde{y}^{(s)}_n \sim \mathcal {N}\!\Big (a^{(s)}\tilde{y}^{(s)}_{n-1},\,q^{(s)}\Big ), $$

where

$$ a^{(s)} = e^{-\theta ^{(s)}\Delta t}, \qquad q^{(s)} = \frac{(\sigma ^{(s)})^2}{2\theta ^{(s)}}\bigl (1-(a^{(s)})^2\bigr ). $$

**Fig. 3:**
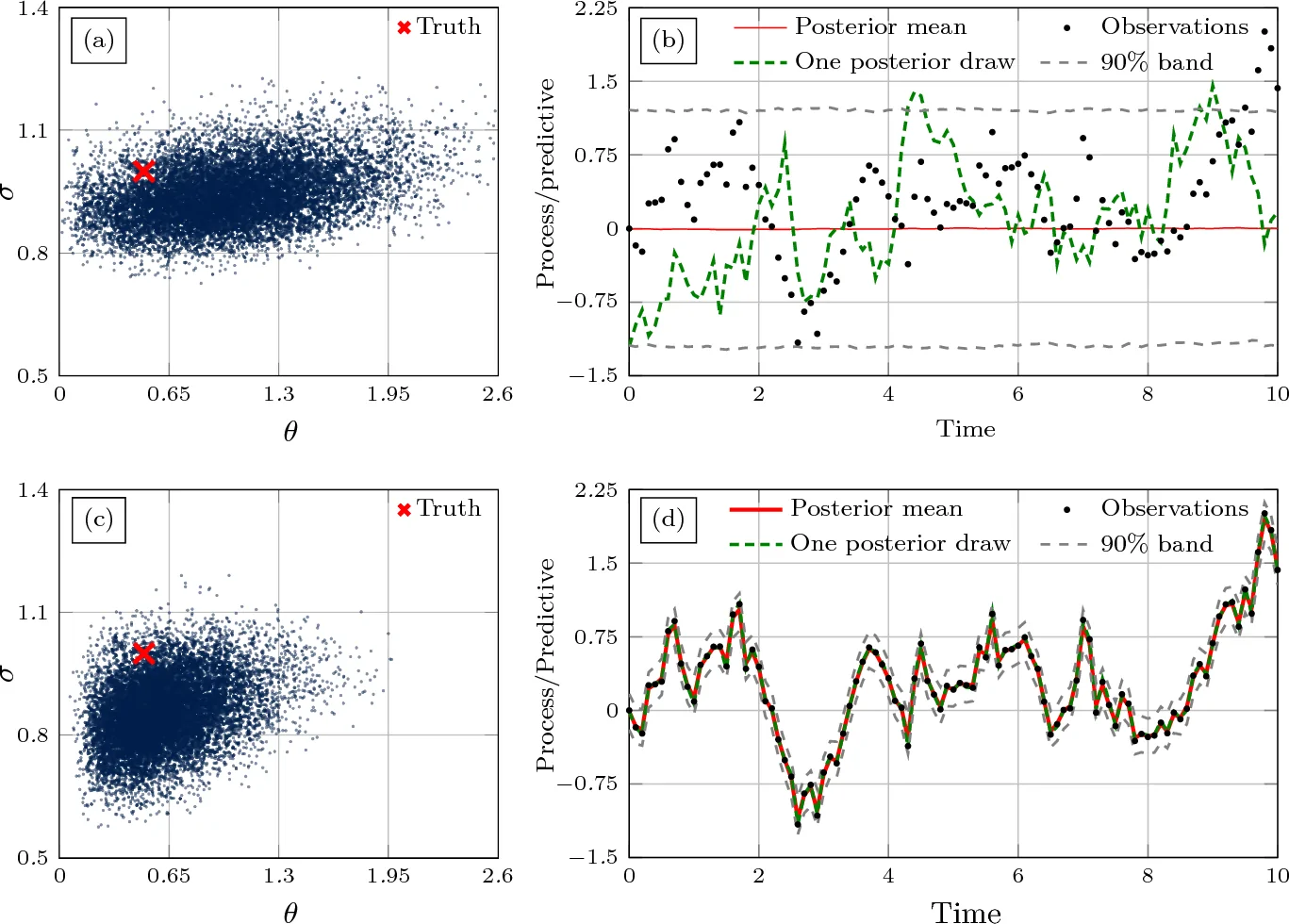
Comparison of OU inference under (a,c) the exact marginal likelihood formulation and (b,d) the state-augmented non-centered formulation (color figure online)

Note that these predictive trajectories evolve independently of the observed series after the initial draw, since they are generated using the simulated predecessor $\tilde{y}_{n-1}$ rather than the realized value $y_{n-1}$. Posterior predictive trajectories generated in this way represent new realizations of the process and therefore do not necessarily follow the observed series pointwise.

In practice, these trajectories are drawn from the posterior predictive distribution

$$ p(\tilde{y} \mid y) = \int p(\tilde{y} \mid \theta ,\sigma )\,p(\theta ,\sigma \mid y)\,d\theta \,d\sigma , $$

which averages over parameter uncertainty but is not conditioned on the realized trajectory itself.

**Case B: Latent Linear–Gaussian State-Space Formulation.**

**Fig. 4:**
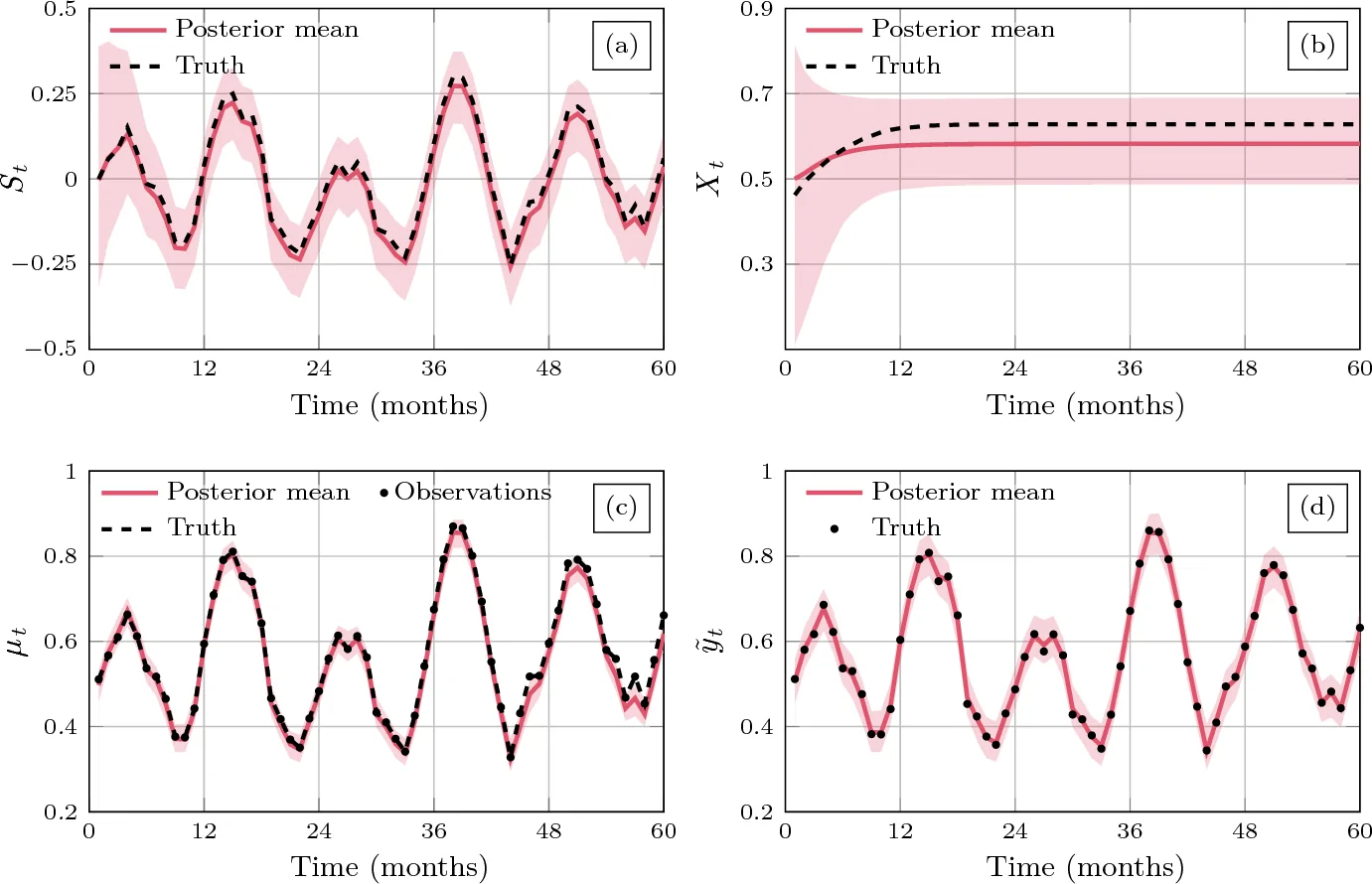
Posterior inference for the latent states in the synthetic case. Shaded regions show 90% credible intervals and solid lines denote posterior means. (a) Deterministic component $X_t$. (b) Stochastic seasonal component $S_t$. (c) Combined signal $\mu _t = X_t + S_t$. (d) Posterior predictive observations $\tilde{y}_t$, compared to the simulated data (color figure online)

In the second formulation the latent states are introduced explicitly and estimated jointly with the parameters. Using the same transition parameters $a=e^{-\theta \Delta t}$ and $q=\frac{\sigma ^2}{2\theta }(1-a^2)$, we construct the states using a non-centered parameterization (Papaspiliopoulos et al. 2007; Betancourt and Girolami 2015).

Let

$$ \eta _0 \sim \mathcal {N}(0,1), \qquad z_n \sim \mathcal {N}(0,1). $$

The latent trajectory is then generated through the recursion

10 $$\begin{aligned} X_1 = \frac{\sigma }{\sqrt{2\theta }}\eta _0, \qquad X_n = a X_{n-1} + \sqrt{q}\,z_{n-1}, \quad n=2,\dots ,N. \end{aligned}$$

The observation model is

11 $$\begin{aligned} y_n \mid X_n,\sigma _e \sim \mathcal {N}(X_n,\sigma _e^2). \end{aligned}$$

**Fig. 5:**
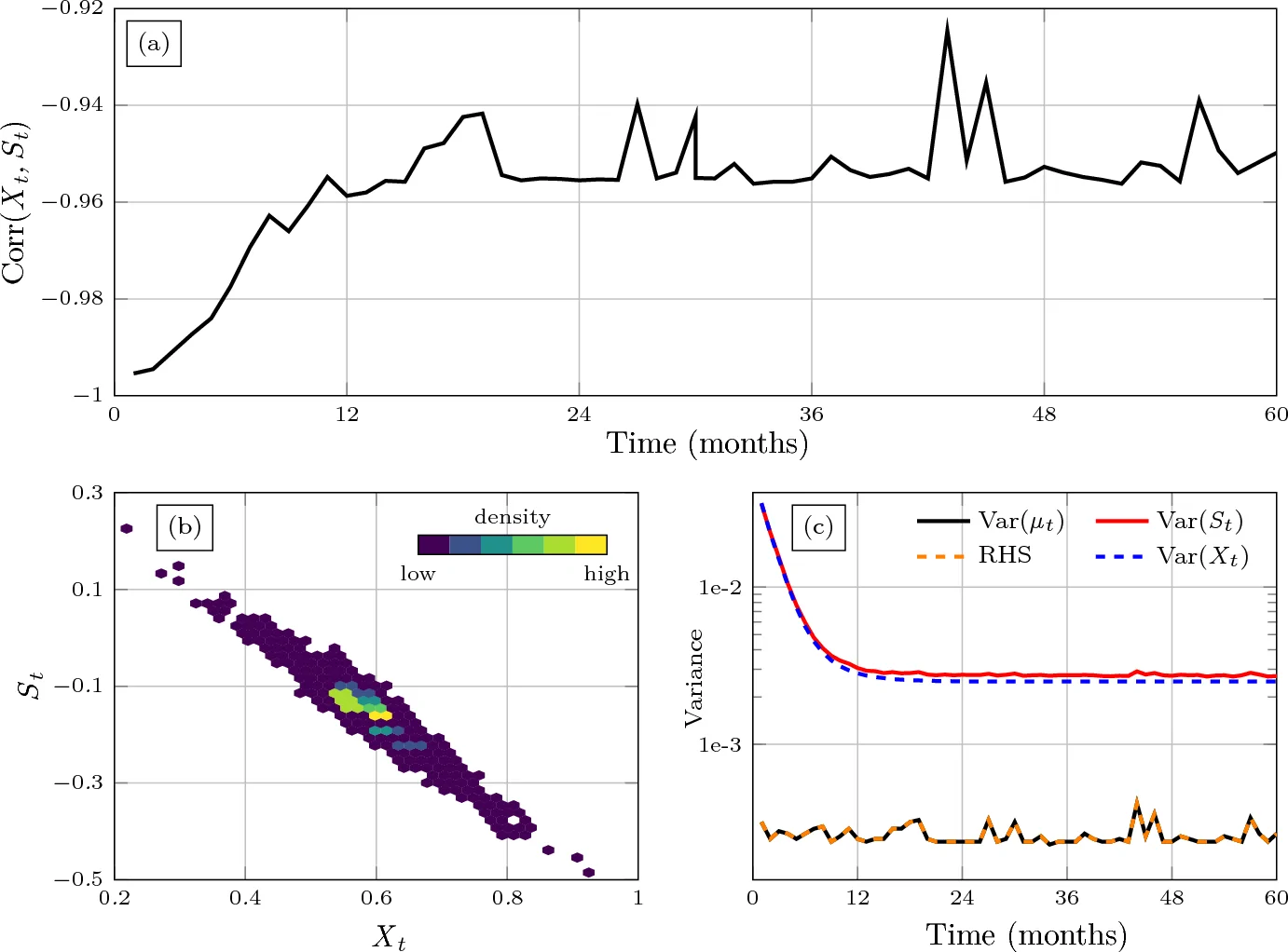
Posterior dependence and variance structure between $X_t$ and $S_t$. (a) Posterior correlation $\textrm{corr}(X_t,S_t)$ over time. (b) Joint posterior at t=30. (c) Variance decomposition over time: $\textrm{Var}(\mu _t)$ and the right-hand side (RHS) of the variance identity (color figure online)

With priors $p(\theta ,\sigma ,\sigma _e)$ and standard-normal priors on $\eta _0$ and $z_{1:N-1}$, the joint density factorizes as

$$\begin{aligned} p(y,X,\eta _0,z,\theta ,\sigma ,\sigma _e)&= \Big [\prod _{n=1}^{N} \mathcal {N}(y_n\mid X_n,\sigma _e^2)\Big ] \, \mathcal {N}(\eta _0\mid 0,1) \\&\prod _{n=1}^{N-1} \mathcal {N}(z_n\mid 0,1) \, p(\theta ,\sigma ,\sigma _e), \end{aligned}$$

where the states $X_{1:N}$ are deterministic functions of $(\eta _0,z_{1:N-1},\theta ,\sigma )$ through (10).

Posterior sampling therefore jointly targets $(\theta ,\sigma ,\sigma _e,\eta _0,z_{1:N-1})$, with each draw inducing a corresponding latent trajectory $X_{1:N}$. Recall that the non-centered parameterization reduces posterior dependence between the innovation scale and the latent states, improving sampling efficiency in Hamiltonian Monte Carlo (Papaspiliopoulos et al. 2007; Betancourt and Girolami 2015).

**Simulation Setup.** To illustrate the two formulations, we generated a synthetic OU trajectory on the interval [0,10] with time step Δt=0.1. The data were simulated using parameter values

**Fig. 6:**
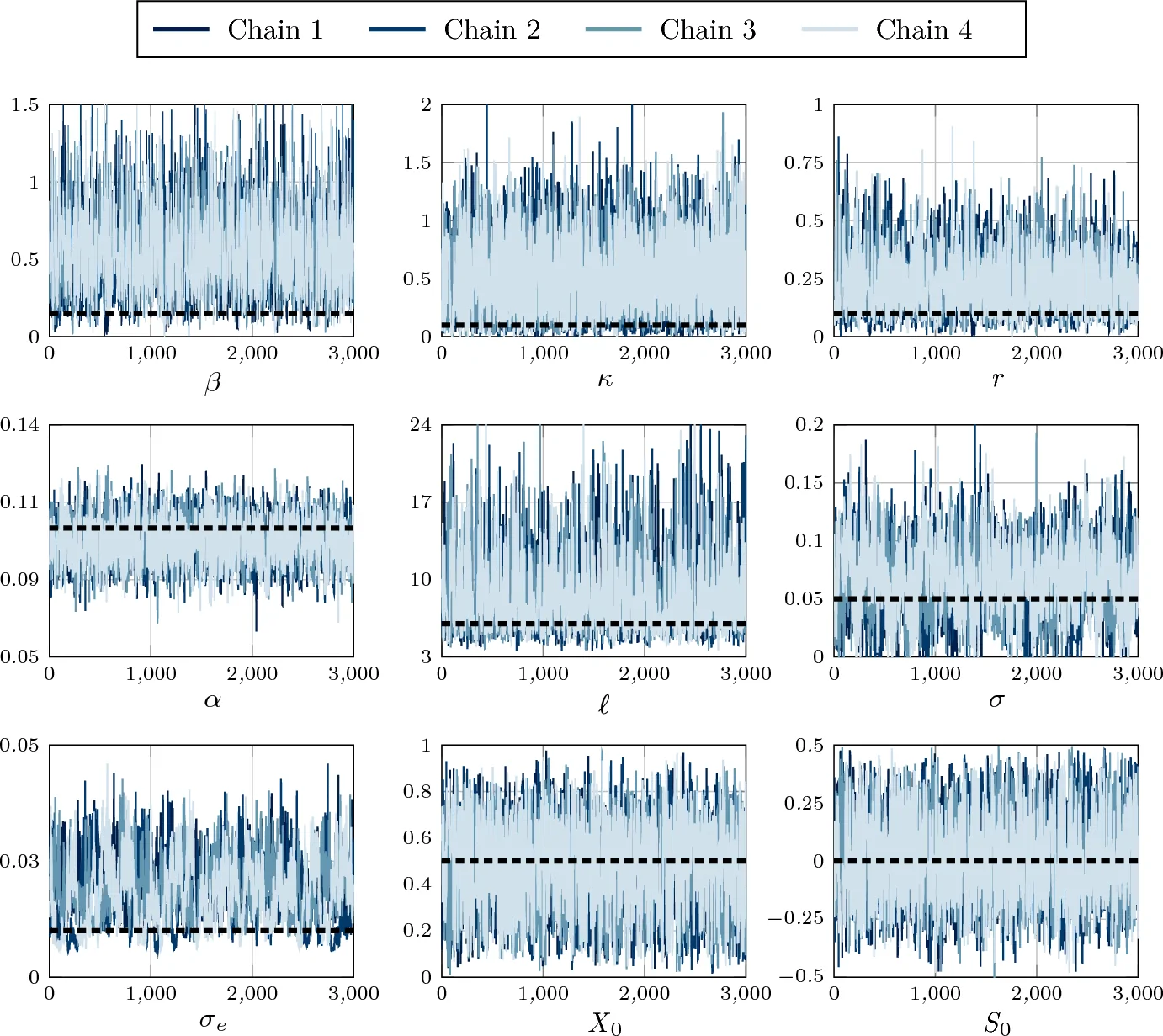
Posterior diagnostics for the synthetic case: trace plots showing the sampled values of parameters over 4 independent MCMC chains. Good mixing is indicated by the chains overlapping and exploring the same region of the parameter space. Dashed horizontal lines indicate the true generating parameter values

**Fig. 7:**
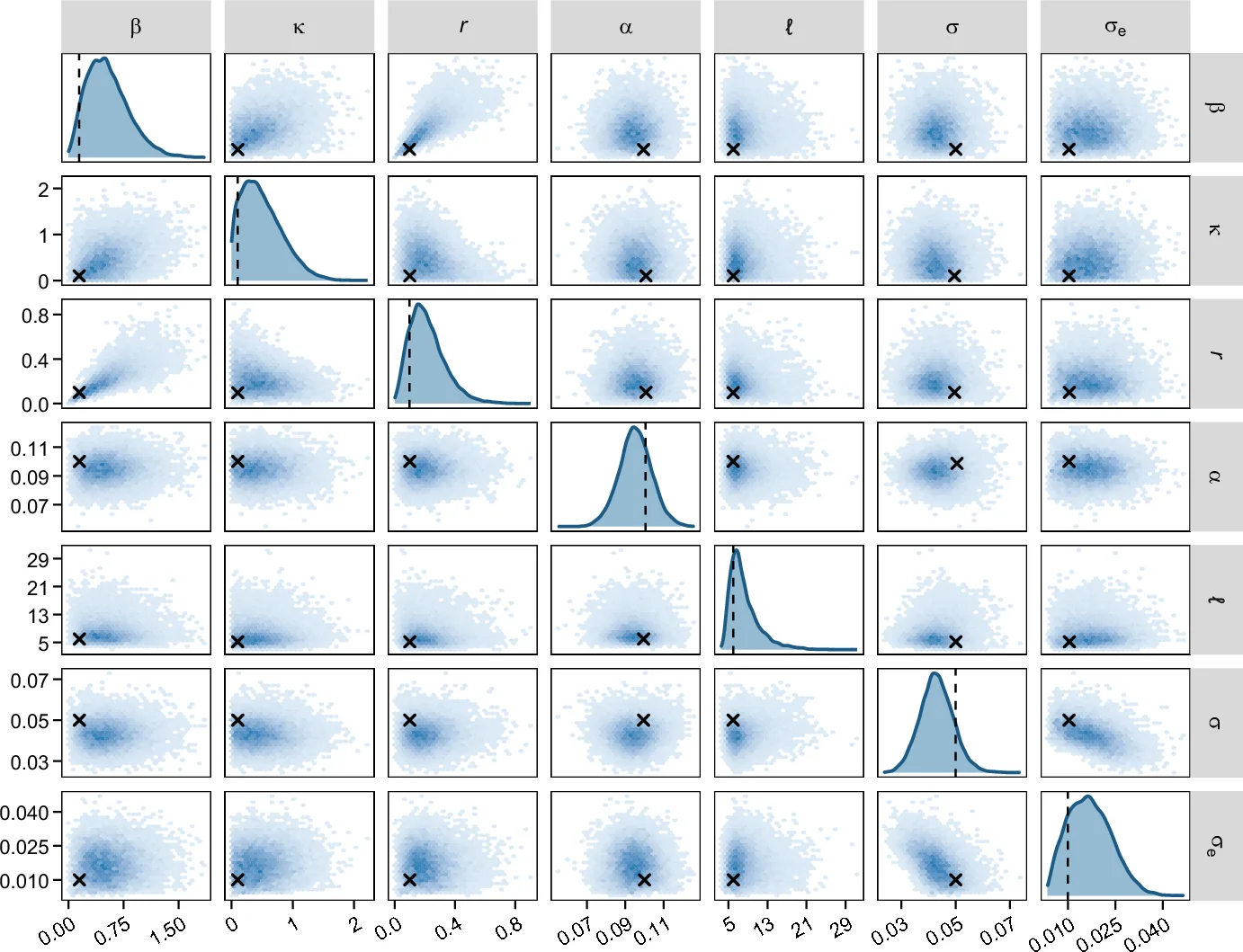
Posterior diagnostics for the synthetic case: pairwise posterior scatter plots for selected parameters, illustrating residual correlations and confirming that the chains converge to a common distribution. Dashed vertical lines and × indicate the true generating parameter values (color figure online)

$$ \theta _{\text {true}}=0.5, \qquad \sigma _{\text {true}}=1.0, $$

and, unless otherwise stated, noiseless observations were assumed ($\sigma _e=0$). Both formulations were then fitted to the simulated data using Bayesian inference.

**Fig. 8:**
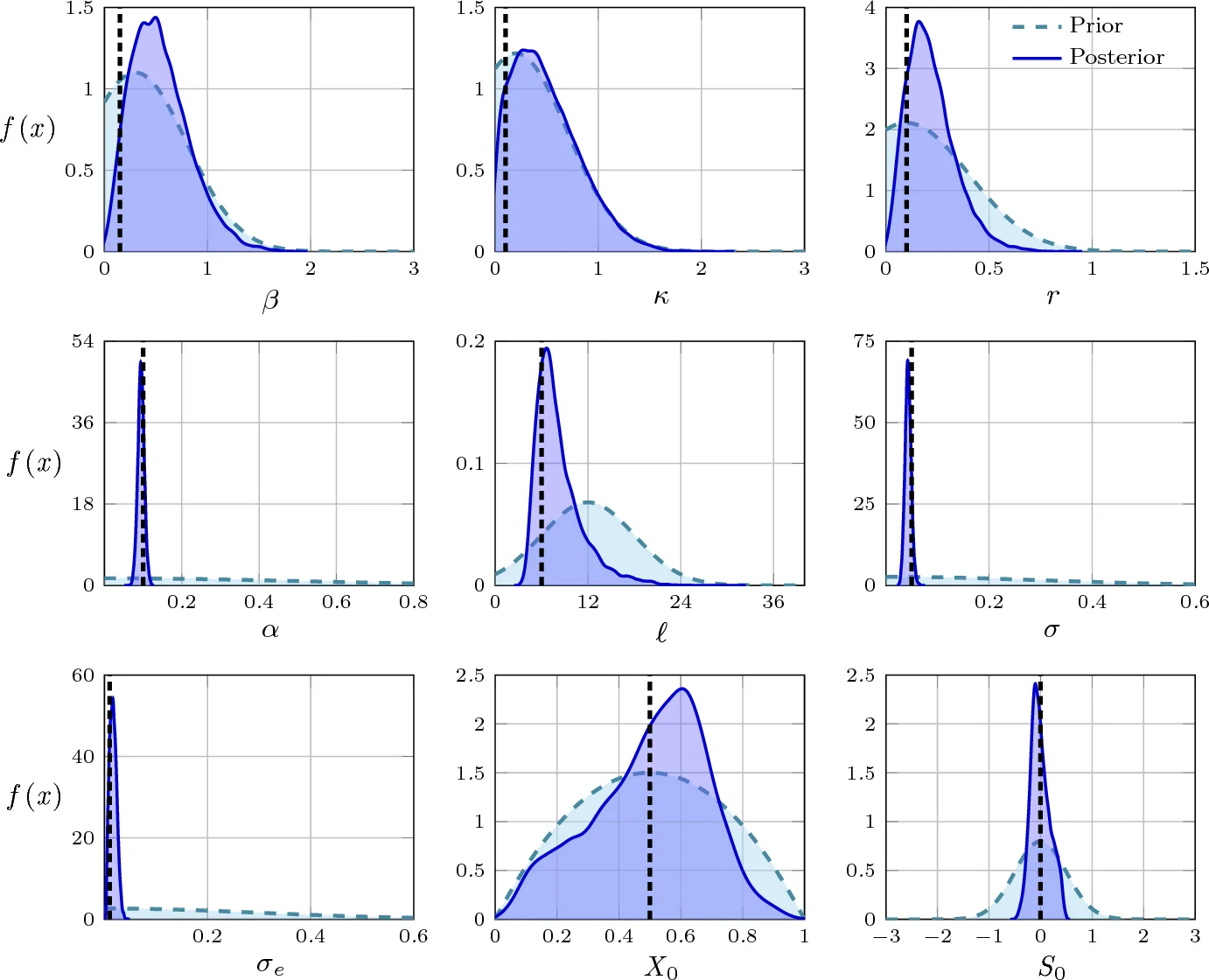
Comparison of prior (dashed) and posterior (solid) distributions for all model parameters. Dashed vertical lines indicate the true generating parameter values (color figure online)

## Results

### OU Validation

Figure 3 compares posterior inference under the two formulations described in Sect. 2. Both approaches recover the parameters $(\theta ,\sigma )$ accurately, as seen in the posterior pair plots.

The trajectory behaviour differs because the two formulations generate different predictive objects. In Case A, posterior predictive paths are drawn unconditionally from $p(\tilde{y} \mid \theta ,\sigma )$. Since each replicate evolves using its own simulated noise sequence, these trajectories represent possible realizations of a new experiment and therefore do not necessarily follow the observed series pointwise.

In contrast, Case B samples latent states jointly with the parameters, yielding draws from the conditional distribution p(X∣y). Predictive trajectories generated from these sampled states therefore remain close to the observed path, particularly when the observation noise is small.

This comparison illustrates why joint parameter–state estimation is useful in stochastic differential equation models: while parameters determine the statistical structure of the process, individual trajectories depend on the underlying noise realizations. When latent states are inferred from the data, the resulting state-conditioned reconstructions naturally align with the observed dynamics. This example also confirms that the non-centered state-space formulation recovers the correct parameters while producing trajectories consistent with the observed realization.

**Fig. 9:**
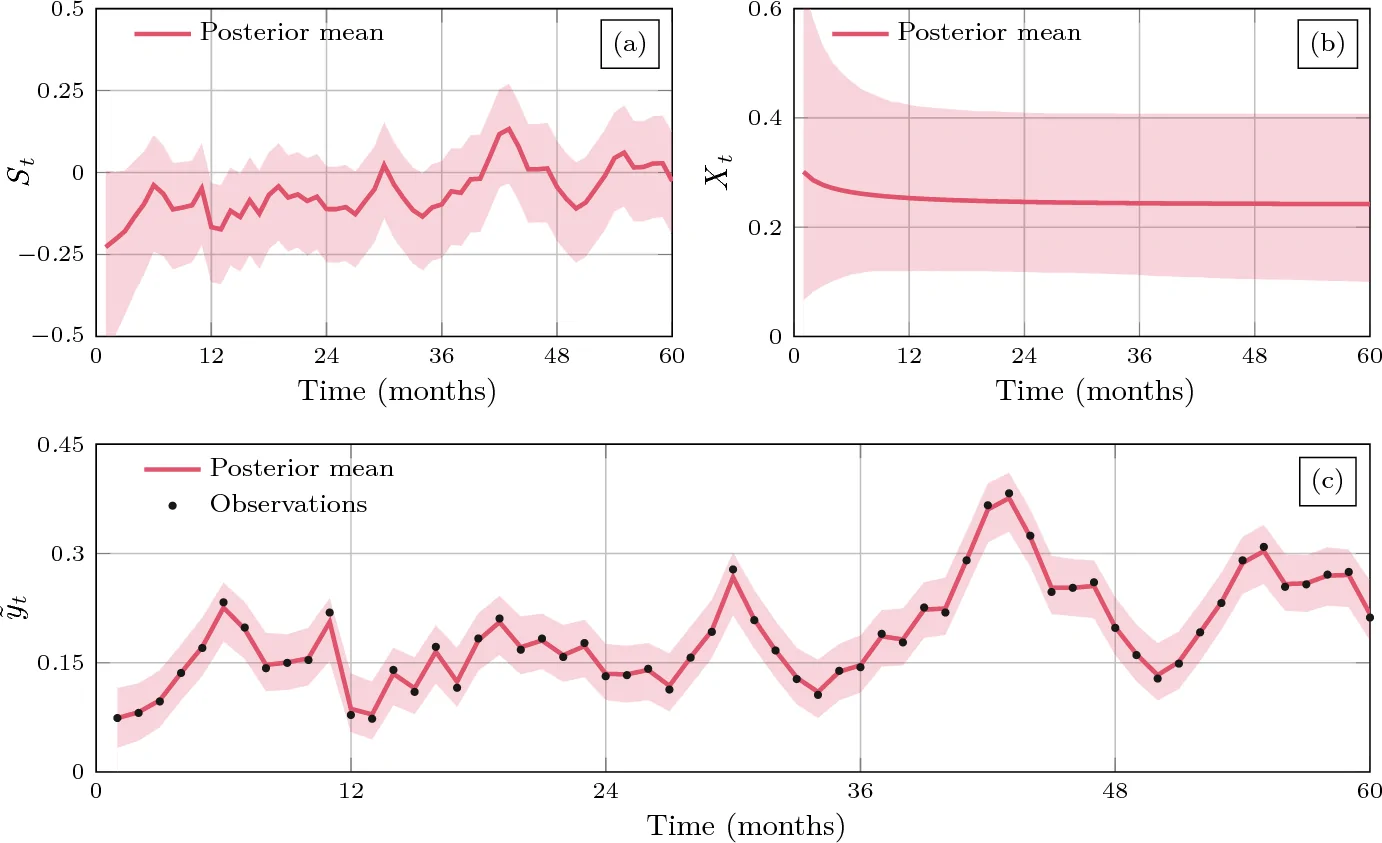
Posterior inference for the real data case. Shaded regions show 90% credible intervals and solid lines denote posterior means. (a) Deterministic component $X_t$. (b) Stochastic seasonal component $S_t$. (c) Posterior predictive observations $\tilde{y}_t$ compared to the reported data (color figure online)

**Fig. 10:**
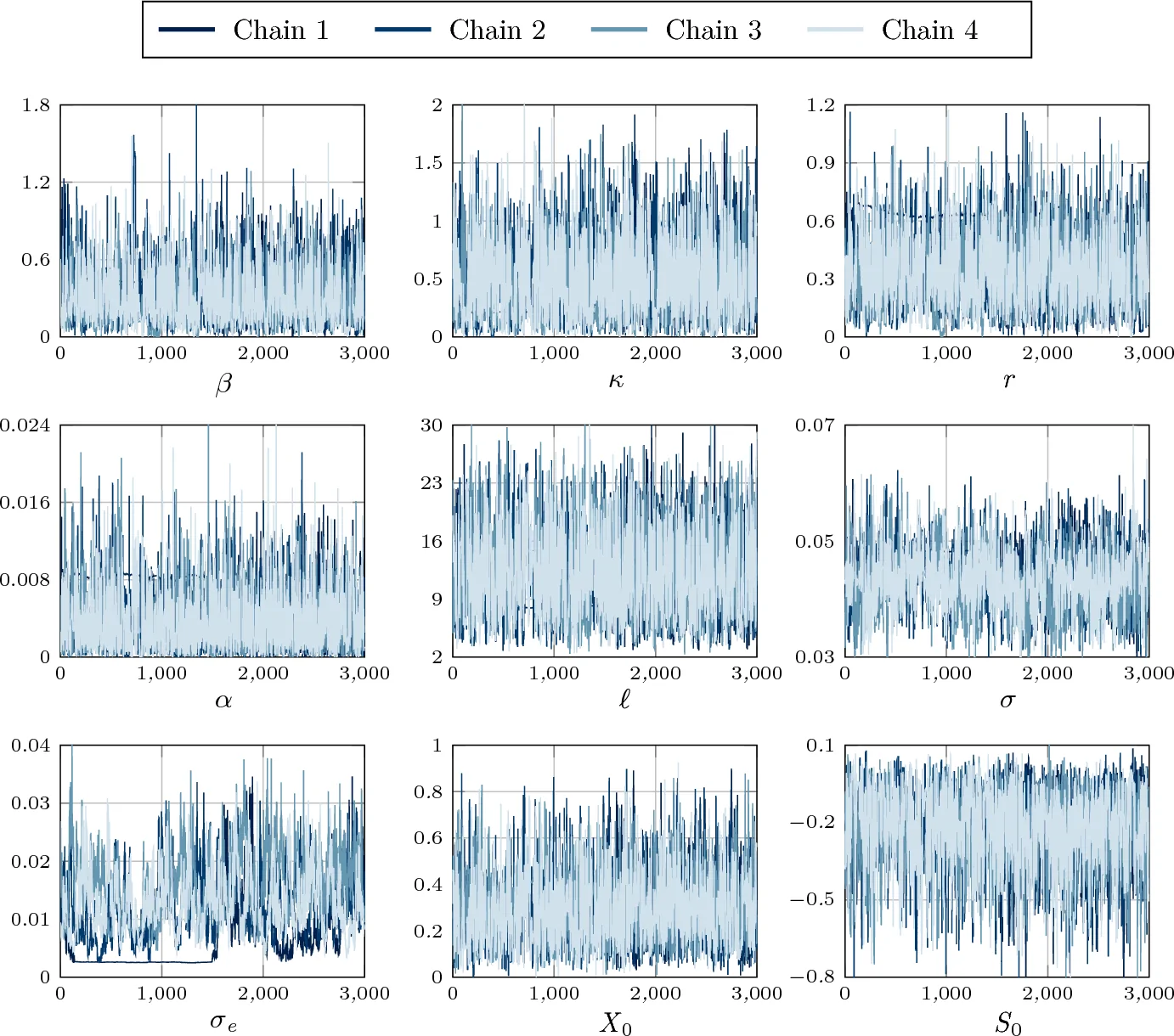
Posterior diagnostics for the real data case: trace plots showing the sampled values of parameters over 4 independent MCMC chains. Good mixing is indicated by the chains overlapping and exploring the same region of the parameter space (color figure online)

Additional validation of the stochastic seasonal component in isolation is provided in Appendix C.

### Synthetic Data Validation

To verify that the additive ODE–SDE formulation behaves as expected under controlled conditions, we first analyse synthetic data generated from the model itself. This experiment allows us to assess whether the model can recover the simulated dynamics, how the deterministic and stochastic components are separated in the posterior, and whether the resulting inference procedure is numerically stable before moving to the real malaria dataset. The synthetic dataset was generated from the additive ODE–SDE model using fixed true parameter values. The ODE parameters were set to β=0.15, κ=0.10, and r=0.10, while the stochastic component parameters were α=0.10, ℓ=6, and σ=0.05, with observation noise $\sigma _e = 0.01$. The initial states were $X_0 = 0.5$ and $S_0 = 0$.

#### Posterior Decomposition and Predictive Performance

Figure 4 shows the posterior means and 90% credible intervals for the latent states and their sum using synthetic data. The additive model reproduces the simulated observations closely: the posterior mean of $\mu _t = X_t + S_t$ follows the data with narrow credible bands. By contrast, the individual components $X_t$ and $S_t$ exhibit wider uncertainty.

The model captures both the long-term trend and seasonal fluctuations in the synthetic data. While the individual components remain uncertain, their sum $\mu _t$ is tightly constrained. Note that these results were obtained using informative priors. The role of prior specification is examined below.

#### Identifiability and Posterior Dependence

Figure 5 illustrates the posterior dependence between the deterministic and stochastic components, $X_t$ and $S_t$. At each time point *t*, the correlation $\textrm{corr}(X_t, S_t)$ was computed across posterior draws. The correlations (panel a) range between -0.99 and -0.92, indicating that the two components move almost perfectly in opposite directions.

Panel (b) shows the joint posterior of $(X_t,S_t)$ at a representative time point (t=30). The samples lie along a narrow negatively sloped ridge, demonstrating that many combinations of $(X_t,S_t)$ produce nearly identical total signals $\mu _t = X_t + S_t$. This negative correlation arises because the two components compete to explain the same observations: increases in one component must be compensated by decreases in the other in order to preserve the total signal. This pattern is typical of additive models in which the total signal is well identified but its internal decomposition is not.

Panel (c) illustrates this relationship using the variance identity

$$ \textrm{Var}(\mu _t) = \textrm{Var}(X_t) + \textrm{Var}(S_t) + 2\,\textrm{Cov}(X_t,S_t), $$

showing that fluctuations in $\textrm{Var}(X_t)$ and $\textrm{Var}(S_t)$ are offset by their large negative covariance, keeping $\textrm{Var}(\mu _t)$ relatively stable. Thus, strong posterior dependence affects the internal decomposition, but not the identification of the total signal.

This strong negative dependence highlights an intrinsic identifiability limitation in decomposing $\mu _t$ into deterministic and stochastic components. Despite this, the combined signal $\mu _t$ remains well identified. Additional experiments with alternative prior specifications confirm that while the individual components $X_t$ and $S_t$ may vary across prior choices, the combined trajectory $\mu _t$ remains essentially unchanged (Appendix D).

#### Posterior Diagnostics

Sampling was performed using Stan’s NUTS algorithm with four chains and 4,000 iterations per chain, of which the first 1,000 were used for warm-up and adaptation. This yielded 3,000 post–warm-up draws per chain (12,000 total).

All parameters achieved $\hat{R} < 1.01$, the potential scale reduction factor used to assess convergence of multiple Markov chains (Gelman and Rubin 1992; Vehtari et al. 2021). Effective sample size (a measure of how many independent draws the correlated MCMC samples are equivalent to) exceeded 400, indicating good chain mixing and reliable posterior estimation. Figures 6 and 7 show representative trace and pairwise plots for selected parameters, demonstrating stable chain behaviour and weak residual correlations.

#### Prior Specification

We first fitted the model to the synthetic dataset using weak (flat) priors with only positivity constraints. In this setting, the parameters were generally poorly identified, and the latent components $X_t$ and $S_t$ were highly uncertain and did not individually track the simulated trajectories.

Among the parameters, those associated with the stochastic component $(\alpha ,\ell ,\sigma )$ and the observation noise $\sigma _e$ were relatively better constrained, while the transmission parameters $(\beta ,\kappa ,r)$ remained weakly identified.

Despite this, the combined signal $\mu _t = X_t + S_t$ remained reasonably well constrained, albeit with substantially wider uncertainty bands. This reflects the identifiability limitation of the additive formulation, where the total signal is informed by the data but its decomposition is not uniquely determined.

To address this, we adopt informative priors that regularise the parameter space and stabilise the decomposition. For the baseline ODE parameters $(\beta ,\kappa ,r)$, prior locations were guided by biologically plausible values derived from malaria transmission parameters reported in the literature (Wallace et al. 2014). Since these parameters are combinations of underlying biological quantities, the literature values were used to define reasonable ranges rather than strict bounds. The priors for the stochastic component $(\alpha ,\ell ,\sigma )$ and the observation noise $\sigma _e$ were chosen to ensure numerical stability. Positivity constraints were imposed on all parameters, and the full specification is given in Table 1.

Figure 8 compares the prior (dashed) and posterior (solid) distributions. The strongest updating is observed for the seasonal and noise parameters: α, ℓ, σ, and $\sigma _e$ show more concentrated posteriors relative to their priors. The transmission parameters β and *r*, as well as the initial states $X_0$ and $S_0$, show moderate updating, while the posterior for κ remains close to its prior, indicating weak information from the data. Overall, the seasonal and noise parameters are more strongly constrained than the baseline transmission parameters. This is plausible, since the stochastic seasonal component captures deviations from the smooth ODE trajectory, including seasonal oscillations and stochastic fluctuations, whereas the deterministic transmission parameters govern the slower underlying dynamics.

Table 1 below.

**Table 1:** Prior distributions for model parameters; $\mathcal {N}^+$ denotes a Normal truncated to (0,∞)

β	$\sim \mathcal {N}^+(0.3,\,0.5)$
κ	$\sim \mathcal {N}^+(0.2,\,0.5)$
*r*	$\sim \mathcal {N}^+(0.1,\,0.3)$
α	$\sim \mathcal {N}^+(0,\,0.5)$
ℓ	$\sim \mathcal {N}^+(12,\,6)$
σ	$\sim \mathcal {N}^+(0,\,0.3)$
$X_0$	$\sim \textrm{Beta}(2,\,2)$
$S_0$	$\sim \mathcal {N}(0,\,0.5)$
$\sigma _e$	$\sim \mathcal {N}^+(0,\,0.3)$

### Real Data Application

We applied the proposed additive model to the same malaria case-report dataset used in our earlier study (Amadi and Haario 2023), consisting of five years (2014-2018) of monthly hospital case records from Delta State, Nigeria (obtained from the National Malaria Elimination Programme, Federal Ministry of Health, Nigeria). Figure 9 summarizes the latent trajectories. The same prior specifications as in Table 1 were used for this case.

The stochastic seasonal component $S_t$ fluctuates around zero with relatively wide credible bands, capturing short-term variation and seasonal effects not represented in the deterministic model. In contrast, the ODE component $X_t$ evolves more smoothly, gradually declining from its initial value before stabilizing.

The posterior predictive observations closely follows the observed case reports, with reasonable posterior credible intervals. Thus, like in the synthetic case, although the individual latent components remain uncertain, their sum is tightly constrained by the data and provides a good representation of the observed dynamics.

Our earlier work (Amadi and Haario 2023) represented seasonal variability through a time-varying mosquito density within a full Ross-type transmission model. The present formulation instead captures unresolved variability through a stochastic seasonal process. Despite these conceptual differences, both approaches recover similar large-scale patterns in the observed case series, suggesting that the additive ODE-SDE framework provides a flexible alternative for representing seasonal forcing and structural model error.

Posterior diagnostics for the real-data fit are shown in Fig. 10. Trace plots show stable sampling behaviour and good mixing across chains.

## Discussion and Conclusion

This study introduced a Bayesian state-space framework that combines deterministic and stochastic components to account for structural bias in simplified dynamical models. By augmenting a nonlinear ODE with a stochastic seasonal process, the method provides a principled way to represent misspecified dynamics within a unified probabilistic formulation. The resulting additive structure links mechanistic and statistical modelling: the deterministic ODE captures the large-scale process dynamics, while the stochastic SDE absorbs residual variability that would otherwise appear as systematic model error.

Inference was performed using Hamiltonian Monte Carlo in Stan, enabling efficient joint estimation of parameters and latent states. The introduction of informative priors was essential to achieve parameter identifiability and numerical stability. Such regularization is common in hierarchical and state–space models, where latent components may otherwise compete to explain the same observations (Gelman et al. 1995; Durbin and Koopman 2012). In the present formulation, the priors constrain the decomposition between deterministic and stochastic effects without restricting the total signal, allowing inference to remain largely data-driven where information is available.

A key finding of the analysis is the strong negative posterior dependence between the deterministic and stochastic components $X_t$ and $S_t$. This behaviour reflects the intrinsic geometry of additive models, in which multiple internal decompositions can generate nearly identical observations. While the individual components are therefore only weakly identifiable, the combined signal $\mu _t = X_t + S_t$ remains well constrained by the data. This illustrates a general property of additive models for accounting for structural bias: although internal components may be uncertain, the observable trajectory can still be robustly identified.

Additional experiments with alternative positivity-constrained priors confirm this behaviour (Appendix D). Although the inferred parameters and latent components vary across prior specifications, the predictive trajectory $\mu _t$ remains essentially unchanged. This result supports the interpretation that the model reliably captures the observable system dynamics even when the internal decomposition is not unique.

From a statistical perspective, the proposed approach can be viewed as a structured extension of the discrepancy framework of  Kennedy and O’Hagan (2001). In that framework, model discrepancy is typically represented by an unconstrained Gaussian process. Here, the discrepancy term is instead represented by a stochastic differential equation, which introduces temporal structure and physical interpretability while preserving flexibility.

The framework improves inference stability and accounts for structural bias in models that are otherwise too rigid to capture complex system dynamics. It also provides coherent uncertainty quantification that reflects both parameter and process variability. While our demonstrations focused on synthetic and epidemiological examples, the same formulation applies broadly to other dynamical systems where stochastic forcing interacts with deterministic structure, including ecological, climate, and energy-system models.

A limitation of the approach is that the decomposition between the deterministic and stochastic components cannot be uniquely identified from the data alone. Consequently, prior specification plays an important role in stabilizing inference (Gelman et al. 1995; Robert 2007; Garcıa-Merino et al. 2026). This is evident in Fig. 8, where the posterior distributions for β and κ remain close to their respective priors, while those for $X_0$ and $S_0$ show only limited updating. Thus, for these quantities where the data are least informative, the inference is driven by the prior. Robustness to prior misspecification therefore becomes a substantive issue because the prior can influence the reported results, in particular the inferred split between the deterministic and stochastic components. It is therefore important to examine how different levels of prior informativeness affect the posterior behaviour, especially for parameters that are only weakly identified. Specifically, it can be checked how narrow the priors must be to ensure stable inference, and how wide they can be before the posterior becomes unstable or weakly identifiable. This also helps identify which parameters benefit most from informative priors and which can remain more weakly constrained. By gradually widening selected priors and monitoring the resulting posterior behaviour, the minimum level of prior information required to obtain stable and interpretable inference without making the model unnecessarily restrictive, can be determined.

Because of these identifiability limitations, the deterministic and stochastic components should not be interpreted as a unique biological separation of the transmission process. Instead, the framework provides a practical approach for reconstructing the overall disease dynamics while quantifying the associated uncertainty.

The framework can be extended to more complex compartmental models, but such an extension is not automatic. In the present study, the stochastic component was introduced because the baseline model failed to reproduce the observed trajectory of infected humans. For a higher-dimensional system, the same strategy would require identifying where the main model–data mismatch occurs and then introducing the stochastic component at that level. A single discrepancy process may still be sufficient when the dominant mismatch is visible in one observed component. In more complex settings, however, different parts of the system may exhibit different types of mismatch, and the form and placement of the stochastic component would need to be chosen case by case. The method is therefore transferable in principle, but its practical extension depends on a defensible modelling choice of how the discrepancy is represented.

## Figures and Tables

**Fig. 11:**
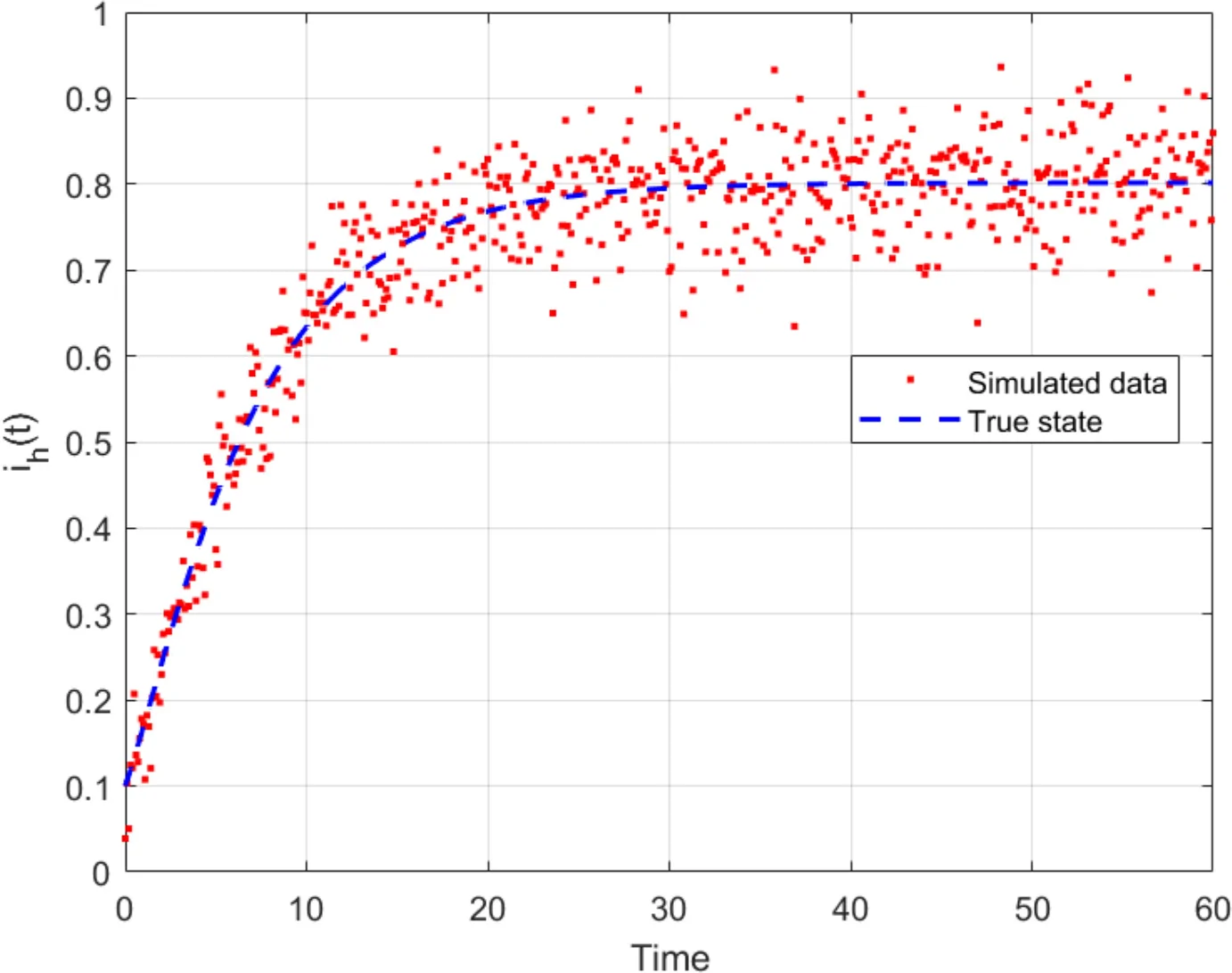
True state trajectory and noisy observations used for the identifiability experiment (color figure online)

**Fig. 12:**
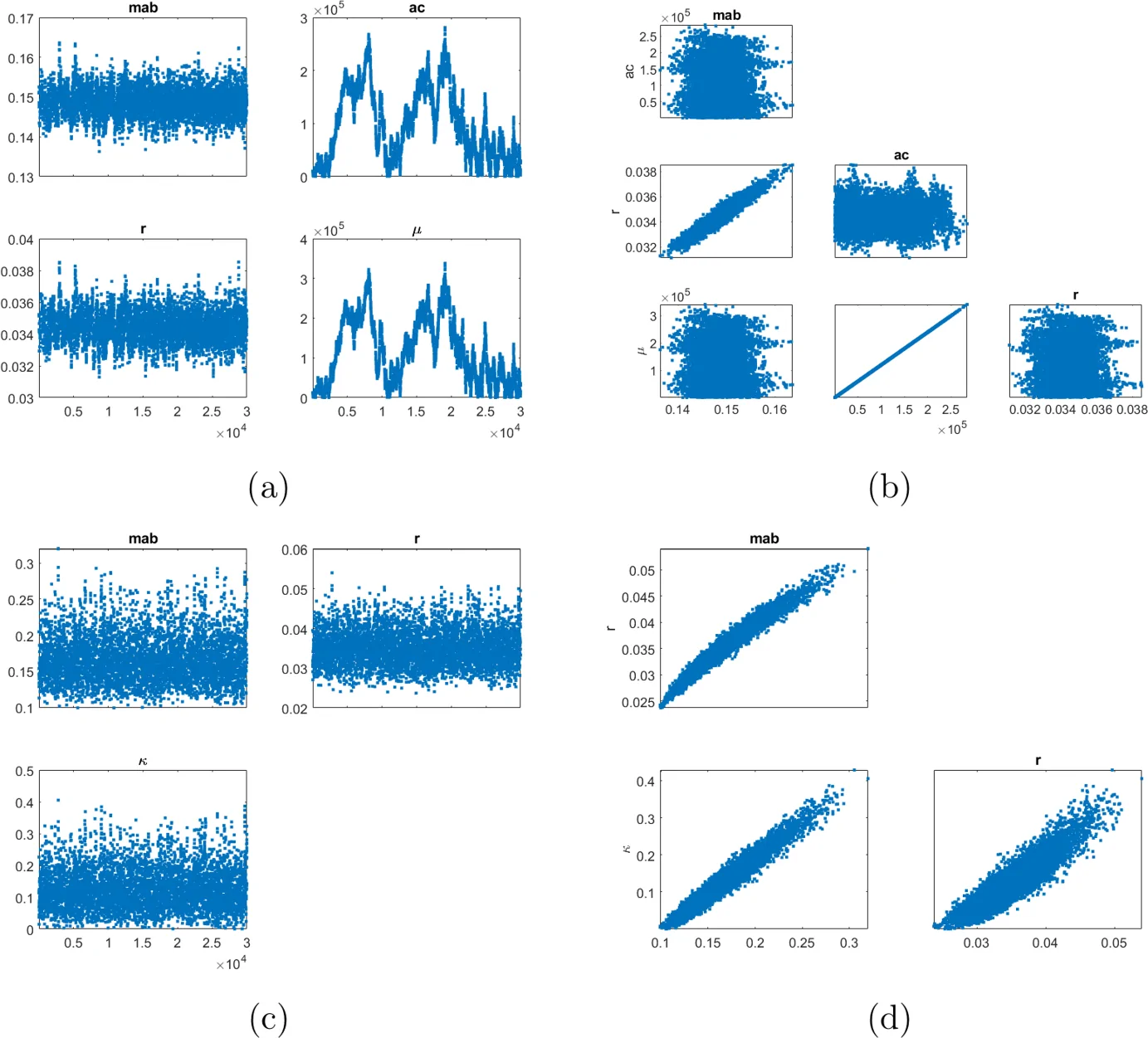
MCMC trace plots (a,c) and pairwise posterior distributions (b,d) for the four-parameter (a,b) and three-parameter (c,d) formulations of the reduced Ross model (color figure online)

**Fig. 13:**
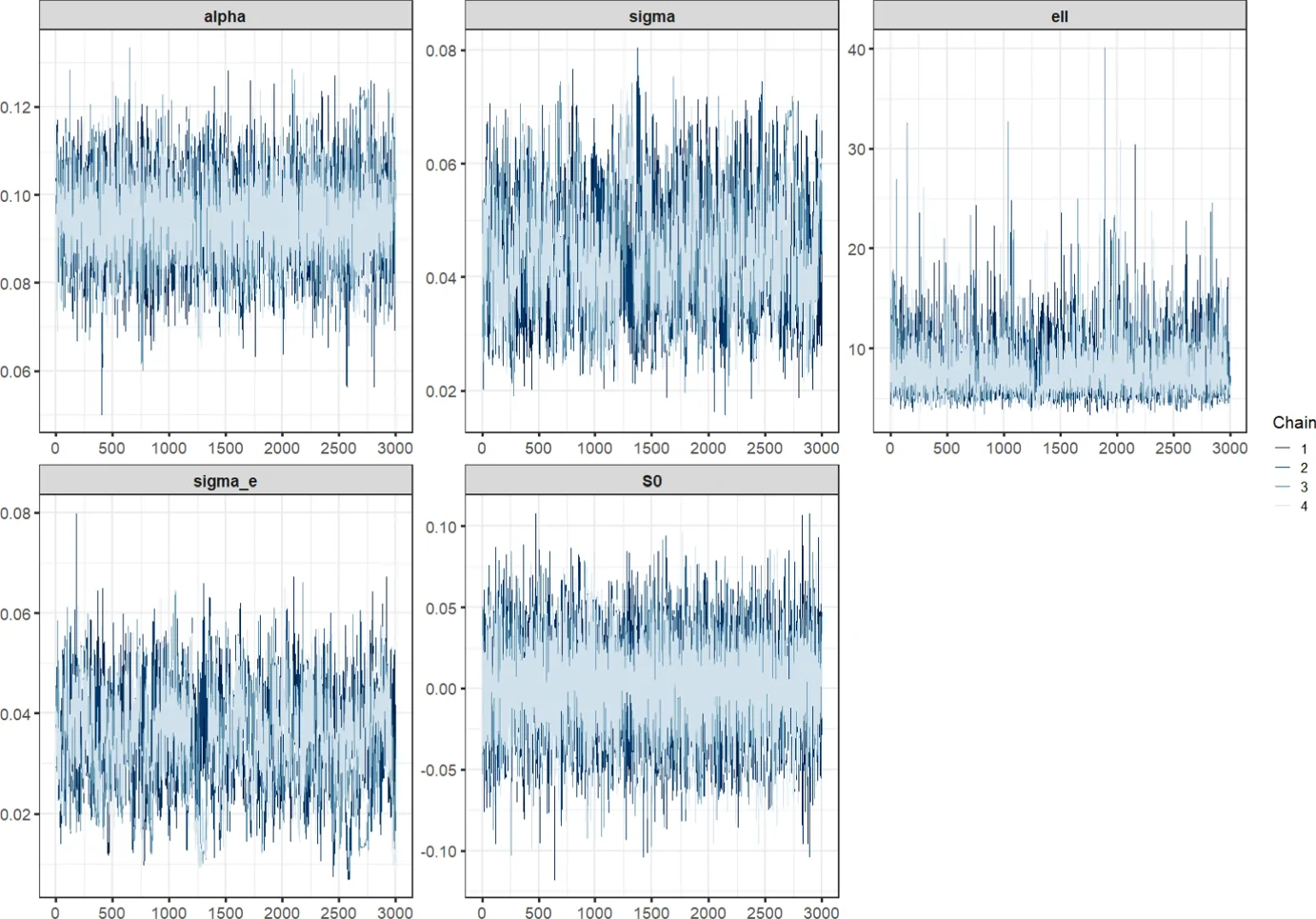
Trace plots for the parameters of the SDE-only model (color figure online)

**Fig. 14:**
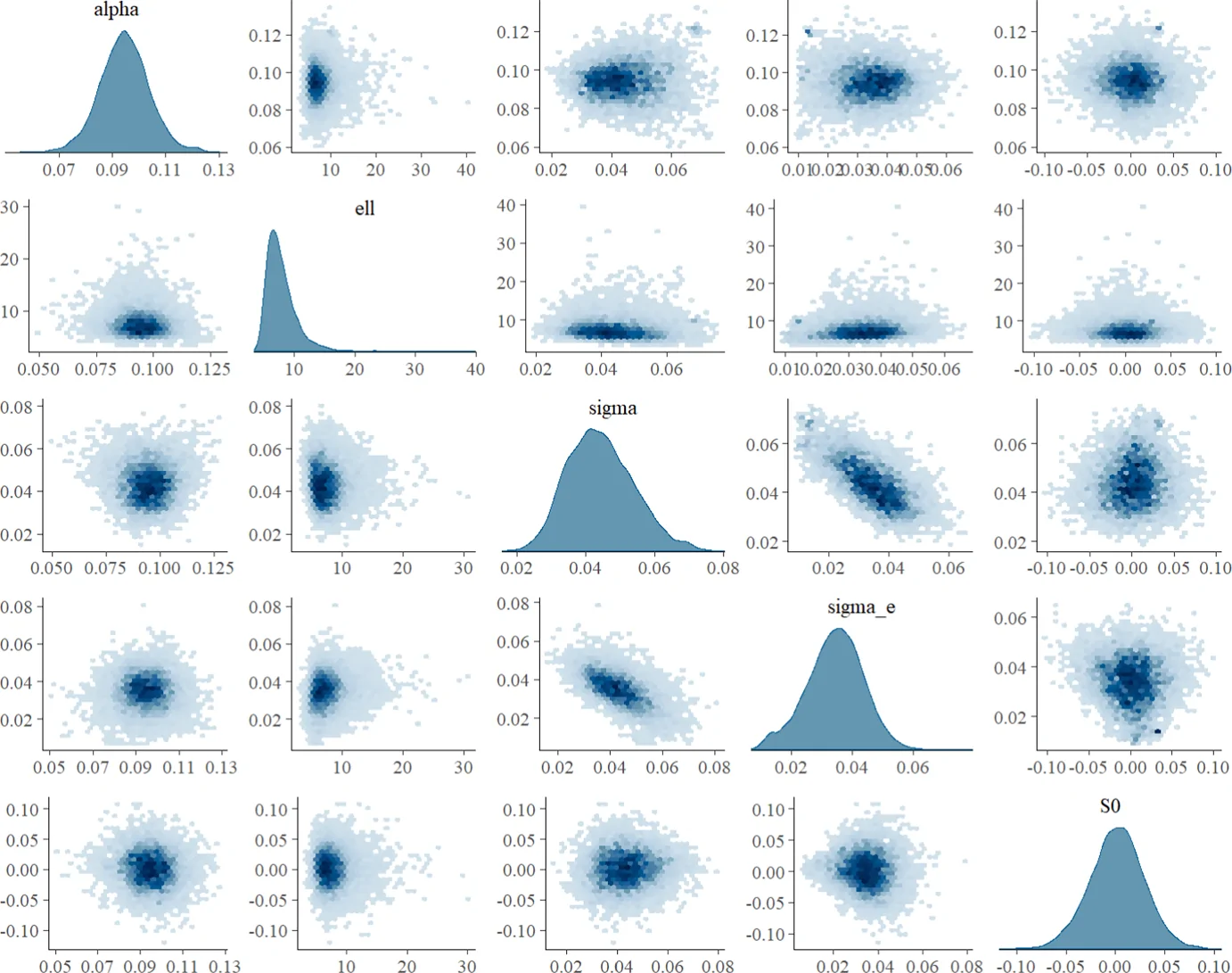
Pairwise posterior densities for the SDE-only parameters $(\alpha ,\ell ,\sigma ,\sigma _e,S_0)$ (color figure online)

**Fig. 15:**
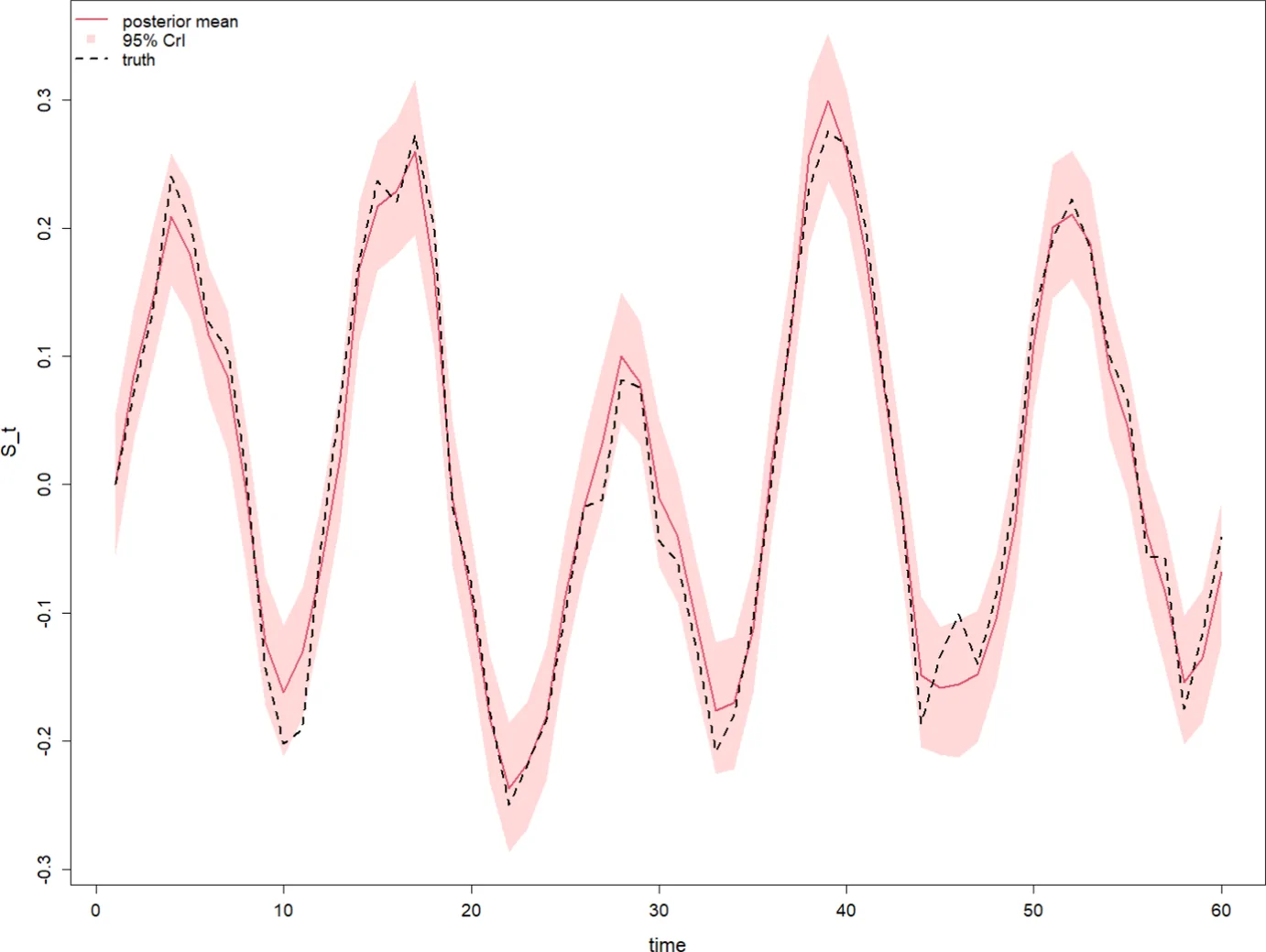
Posterior trajectory fit for the SDE-only model. The red line shows the posterior mean of $S_t$, the shaded region represents the 95% credible interval, and the dashed black line denotes the true simulated trajectory (color figure online)

**Fig. 16:**
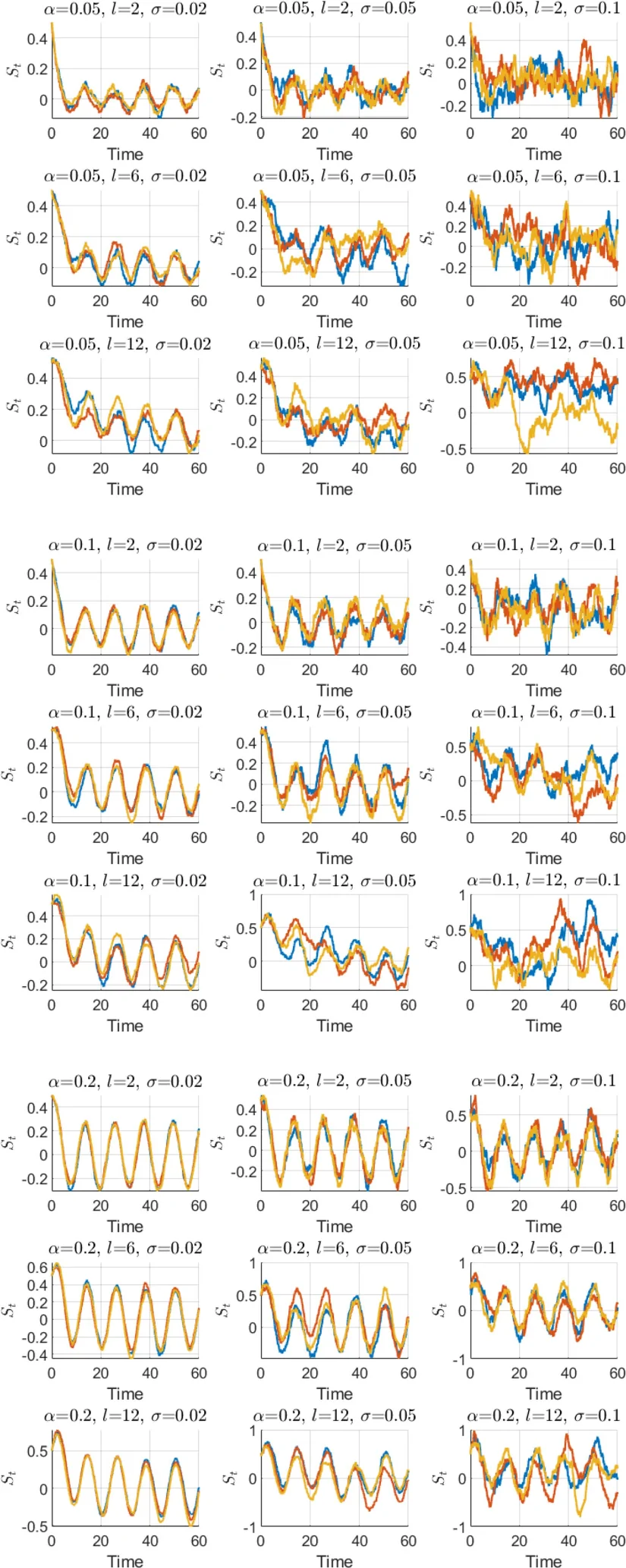
Simulated realizations of the stochastic seasonal process $S_t$ for 27 parameter combinations (color figure online)

## Data Availability

The malaria case data used in this study were obtained from the National Malaria Elimination Programme, Federal Ministry of Health, Nigeria. Due to data-sharing restrictions, the dataset is not publicly available. Data may be made available from the corresponding author upon reasonable request.

## Additional Background Material

### Relation to the Ornstein–Uhlenbeck Process

The classical Ornstein–Uhlenbeck (OU) process satisfies

$$ dS_t = -\tfrac{1}{\ell }(S_t-\mu )\,dt + \sigma \,dW_t, $$

where μ is the equilibrium mean. In this formulation the state is continuously pulled toward μ, which also represents the long-run expectation of the process.

To capture seasonal malaria dynamics we introduce a periodic forcing term,

$$ dS_t = \left( \alpha \cos \!\frac{2\pi t}{T} - \frac{S_t}{\ell }\right) dt + \sigma dW_t. $$

This can be written in OU form as

$$ dS_t = -\frac{1}{\ell }\big (S_t - m(t)\big )dt + \sigma dW_t, $$

with time-varying mean

$$ m(t) = \ell \alpha \cos \!\frac{2\pi t}{T}. $$

Thus the process reverts to an oscillating seasonal mean rather than a constant equilibrium.

Taking expectations removes the stochastic term, giving

$$ \frac{d\mu _t}{dt} = -\frac{1}{\ell }\big (\mu _t-m(t)\big ), $$

whose solution approaches $\mu _t \approx m(t)$ once transient effects decay. The seasonal OU formulation therefore provides a natural stochastic representation of periodic environmental forcing.

### Identifiability of the Reduced Ross Model

We also investigated identifiability of the reduced Ross model using synthetic data generated with true parameter values

$$ [\beta ,\kappa ,r] = [0.15,\,0.1,\,0.033]. $$

Bayesian inference was performed using Markov Chain Monte Carlo sampling with weakly informative priors defined on the positive real line (implemented as improper uniform priors on (0,∞)). Sampling was performed using the adaptive Metropolis algorithm (Haario et al. 2006), which updates the proposal covariance matrix during sampling. The initial proposal covariance was set to $0.01\textbf{I}$.

We compared the four-parameter and three-parameter formulations using synthetic data to determine which parameterisation is better identified (Figs. 11 and 12).

## Bayesian State-Space Formulation of the Stochastic Seasonal Component

Having defined the stochastic seasonal process, we now describe its probabilistic representation as a state–space model. Let $\psi = \{\alpha , \ell , \sigma \}$ denote the SDE parameters.

The Euler–Maruyama discretisation of the SDE yields an approximate Gaussian transition density for the latent process, since the increments of the Wiener process are normally distributed (Oksendal 2013; Kloeden and Pearson 1977). Conditional on the parameters ψ and the previous state $S_{t-1}$, the transition distribution is therefore

12 $$\begin{aligned} S_t \mid S_{t-1},\psi \sim \mathcal {N}\!\left( S_{t-1} + \left( \alpha \cos \!\left( \tfrac{2\pi (t-1)}{T}\right) - \tfrac{S_{t-1}}{\ell }\right) \!\Delta t,\; \sigma ^2 \Delta t \right) . \end{aligned}$$

Equation (12) defines the transition density of the latent state process. Because the state sequence $S_t$ is Markovian, the joint distribution of the state trajectory factorises into a product of transition densities (Durbinand Koopman 2012; West and Harrison 1997):

13 $$\begin{aligned} p(S_{1:N}\mid S_0,\psi ) = \prod _{t=1}^{N} p(S_t\mid S_{t-1},\psi ). \end{aligned}$$

Substituting the Gaussian transition density from (12) into (13) yields

14 $$\begin{aligned} p(S_{1:N} \mid S_0, \psi ) = \prod _{t=1}^{N} \mathcal {N}\!\left( S_t \mid S_{t-1} + \left( \alpha \cos \!\left( \tfrac{2\pi (t-1)}{T}\right) - \tfrac{S_{t-1}}{\ell } \right) \Delta t,\, \sigma ^2\Delta t \right) . \end{aligned}$$

Taking the logarithm of the prior (up to an additive constant) gives

15 $$\begin{aligned} \log p(S_{1:N} \mid S_0, \psi ) = -\frac{1}{2\sigma ^2 \Delta t} \sum _{t=1}^{N} \left( S_t - S_{t-1} - \left( \alpha \cos \left( \tfrac{2\pi (t-1)}{T}\right) - \tfrac{S_{t-1}}{\ell } \right) \Delta t \right) ^2 . \end{aligned}$$

We also specify a prior for the initial state $S_0$, for example

$$ S_0 \sim \mathcal {N}(\mu _0,\sigma _0^2). $$

Thus the full prior over the latent trajectory is

16 $$\begin{aligned} p(S_{0:N} \mid \psi ) = p(S_0)\prod _{t=1}^{N} p(S_t \mid S_{t-1}, \psi ). \end{aligned}$$

Observations $\textbf{y} = (y_1,\dots ,y_N)$ are modeled as noisy versions of the latent states $\textbf{S}_{1:N} = (S_1,\dots ,S_N)$ (Durbin and Koopman 2012):

17 $$\begin{aligned} \textbf{y} = \textbf{S}_{1:N} + \boldsymbol{\epsilon }, \qquad \boldsymbol{\epsilon } \sim \mathcal {N}(\textbf{0},\Sigma ), \end{aligned}$$

where Σ is the observation noise covariance matrix. The general Gaussian likelihood is

18 $$\begin{aligned} \log p(\textbf{y} \mid \textbf{S}_{1:N}) = -\frac{1}{2}\log \det (2\pi \Sigma ) -\frac{1}{2} (\textbf{y}-\textbf{S}_{1:N})^\top \Sigma ^{-1} (\textbf{y}-\textbf{S}_{1:N}). \end{aligned}$$

Under the common assumption of iid observation noise, $\Sigma = \sigma _e^2 I$, the likelihood reduces to

19 $$\begin{aligned} p(\textbf{y} \mid \textbf{S}_{1:N},\sigma _e^2) = \prod _{t=1}^N \mathcal {N}(y_t \mid S_t,\sigma _e^2). \end{aligned}$$

Applying Bayes’ theorem, the posterior distribution over states and parameters is

20 $$\begin{aligned} p(S_{0:N},\psi ,\sigma _e^2 \mid \textbf{y}) = \frac{ p(\textbf{y} \mid \textbf{S}_{1:N},\sigma _e^2) \, p(S_{0:N} \mid \psi ) \, p(\psi ) \, p(\sigma _e^2) }{ p(\textbf{y}) }. \end{aligned}$$

Ignoring the marginal likelihood $p(\textbf{y})$, the posterior is proportional to

21 $$\begin{aligned} p(S_{0:N}, \psi , \sigma _e^2 \mid \textbf{y})&\propto p(\textbf{y} \mid \textbf{S}_{1:N}, \sigma _e^2) \, p(S_{0:N} \mid \psi ) \, p(\psi ) \, p(\sigma _e^2) \end{aligned}$$

22 $$\begin{aligned}&= p(\textbf{y} \mid \textbf{S}_{1:N}, \sigma _e^2) \, p(S_0) \prod _{t=1}^{N} p(S_t \mid S_{t-1}, \psi ) \, p(\psi ) \, p(\sigma _e^2). \end{aligned}$$

## Stochastic Seasonal Component Validation

This appendix provides additional validation of the stochastic seasonal component used in the additive ODE–SDE framework. We first examine the behaviour of the stochastic component in isolation and then illustrate how different parameter combinations influence the resulting seasonal trajectories.

### SDE-Only Model

To verify that the stochastic seasonal process can be recovered independently, we perform joint parameter and state estimation for the SDE component alone. The discretized seasonal process is written as

$$ S_{t} = S_{t-1} + \Delta t \Bigl ( \alpha \cos \!\tfrac{2\pi (t-1)}{T} - \tfrac{S_{t-1}}{\ell }\Bigr ) + \sigma \sqrt{\Delta t}\, z_{t-1}, $$

where the innovations $z_{t-1} \sim \mathcal {N}(0,1)$ are independent standard normal variables. This non-centred representation improves sampler efficiency and reduces posterior correlations between scale parameters and latent states.

Posterior diagnostics (Fig. 13) indicate stable chain behaviour and good mixing. The pairwise posterior densities (Fig. 14) are unimodal and concentrated near the true parameter values, with correlations reflecting the expected trade-off between the process noise σ and observation noise $\sigma _e$.

The trajectory reconstruction (Fig. 15) shows that the posterior mean of $S_t$ closely tracks the simulated realization, while the 95% credible band provides reliable uncertainty quantification. These results demonstrate that the stochastic seasonal component can be recovered in practice and provide a baseline validation before combining it with deterministic ODE dynamics.

### Illustration of Seasonal Process Realisations

To illustrate the effect of parameter choices on seasonal dynamics, we simulated multiple realizations of the stochastic seasonal process (5). The simulations were performed with seasonal period T=12 and parameter values drawn from

$$ \alpha \in \{0.05,\,0.1,\,0.2\}, \qquad \ell \in \{2,\,6,\,12\}, \qquad \sigma \in \{0.02,\,0.05,\,0.1\}. $$

For each parameter combination, three independent trajectories were generated to illustrate the range of possible stochastic behaviours.

Figure 16 shows how different parameter combinations produce qualitatively similar temporal patterns. The parameters interact to control both the amplitude and persistence of seasonal fluctuations. These interactions can lead to practical parameter non-identifiability, motivating the Bayesian inference framework used in the main analysis.

## Prior Specification Variants

Because the proposed model decomposes the signal into two latent components

$$ \mu _t = X_t + S_t, $$

the likelihood cannot uniquely determine how variability should be allocated between them. To examine robustness to alternative prior forms, we compared two reasonable prior specifications.

For the ODE parameters we considered both lognormal and truncated-normal forms, for example

$$ \beta \sim \textrm{LogNormal}(\log 0.3,1.0) \qquad \text {or} \qquad \beta \sim \mathcal {N}(0.3,0.5)\mathbb {I}_{[0,\infty )}. $$

Analogous alternatives were used for κ and *r*. The seasonal amplitude was assigned $\alpha \sim \mathcal {N}^+(0,0.5)$ and the seasonal time scale was centred near the seasonal period *T* via

$$ \ell \sim \textrm{LogNormal}(\log T,1.0) \quad \text {or}\quad \ell \sim \mathcal {N}^+(T,6). $$

The stochastic scale parameter followed $\sigma \sim \mathcal {N}^+(0,0.3)$, while the initial states were specified as

$$ X_0 \sim \textrm{Beta}(2,2), \qquad S_0 \sim \mathcal {N}(0,0.5). $$

Observation noise was assigned $\sigma _e \sim \mathcal {N}^+(0,0.3)$.

Table 2 compares posterior estimates obtained under the two prior variants.

**Table 2:** Posterior means (standard deviations) under two prior specifications

Parameter	Lognormal prior	Half-normal prior
β	0.28 (0.22)	0.56 (0.29)
κ	0.36 (0.54)	0.49 (0.33)
*r*	0.13 (0.09)	0.23 (0.12)
α	0.09 (0.01)	0.09 (0.01)
ℓ	7.41 (4.28)	8.08 (3.35)
σ	0.05 (0.01)	0.05 (0.01)
$\sigma _e$	0.03 (0.01)	0.03 (0.01)
$X_0$	0.52 (0.19)	0.51 (0.19)
$S_0$	-0.02 (0.19)	0.00 (0.18)
RMSE$(X_t)$	0.0216	0.0167
RMSE$(S_t)$	0.0310	0.0276
RMSE$(\mu _t)$	0.0218	0.0218

While individual parameter estimates differ somewhat between specifications, the predictive accuracy of the combined signal $\mu _t$ remains essentially unchanged. This confirms that although the internal decomposition into $X_t$ and $S_t$ is not uniquely identifiable, the observable quantity $\mu _t$ is robustly constrained by the data.
